# Supplementary material for: Does a humoral correlate of protection exist for SARS-CoV-2? A systematic review
Source: PLoS One. 2022 Apr 8;17(4):e0266852. doi: 10.1371/journal.pone.0266852 (PMC8993021; doi:10.1371/journal.pone.0266852)
Supplement: S1 Table — (PDF) [file pone.0266852.s001.pdf]

Public Health Ontario | Santé publique Ontario

LITERATURE SEARCH

08/27/2021

COVID-19 antibody correlates of protection

Request prepared by: Library Services
Contact information: library@oahpp.ca

Search results reporting

DATABASES SEARCHED

Database	Date searched	Records	Duplicates removed by database	Remaining
MEDLINE (I)	08/27/2021	1824	0	1824
MEDLINE (II)	08/27/2021	735	0	735

RESULTS TOTALS

Records source	Records
Records identified through database searching	2559
Duplicates removed by database	0
Duplicates removed by bibliographic management software	101
Total records after duplicates removed	2458

Search strategies

MEDLINE

Ovid MEDLINE(R) ALL <1946 to August 26, 2021>

#	Searches	Results

1	COVID-19 Vaccines/ or (ncov or BNT162* or "mrna-1273").rn,nm,os,ox,ps,px,rs,rx. or ("Ad5-nCoV vaccine" or "BNT162 vaccine" or "ChAdOx1 COVID-19 vaccine" or "Covid-19 aAPC vaccine" or "lentiviral minigene vaccine of COVID-19 coronavirus" or "mRNA-1273 vaccine" or "recombinant SARS-CoV-2 vaccine NVX-cov2373" or "SARS-CoV-2 inactivated vaccines").rn. or (BNT162* or Comirnaty or CoronaVac or Covishield or Tozinameran or "mrna-1273*" or ChAdOx1* or AZD1222 or "Ad26.COV2.S" or "JNJ-78436735" or Novavax or NVX-CoV2373 or Ad5-nCoV or aAPC or "lentiviral minigene" or vaxzevria).ti,kf,kw.	4959
2	COVID-19/ or SARS-CoV-2/ or (Pandemics/ and Coronavirus Infections/) or "SARS-CoV-2 variants".os. or COVID-19 Testing/ or COVID-19 Serological Testing/ or ("COVID-19" or "SARS-CoV-2").nm,os,ox,ps,px,rs,rs. or ("2019 corona virus" or "2019 coronavirus" or "2019 ncov" or "corona virus 19" or "corona virus 2019" or "corona virus disease 19" or "corona virus disease 2019" or "corona virus epidemic*" or "corona virus outbreak*" or "corona virus pandemic*" or "corona virus response" or "coronavirus 19" or "coronavirus 2019" or "coronavirus disease 19" or "coronavirus disease 2019" or "coronavirus epidemic*" or "coronavirus outbreak*" or "coronavirus pandemic*" or "coronavirus response" or "covid 19" or "covid 2019" or "new corona virus" or "new coronavirus" or "novel corona virus" or "novel coronavirus" or "novel human coronavirus" or "sars coronavirus 2" or "sars cov 2" or "sars cov2" or "sars like coronavirus" or "severe acute respiratory syndrome corona virus 2" or "severe acute respiratory syndrome coronavirus 2" or "severe specific contagious pneumonia" or "wuhan corona virus" or "wuhan coronavirus" or ((pandemic* or novel or wuhan) adj3 (coronavirus* or "corona virus*" or betacoronavirus* or "beta coronavirus*" or "beta corona virus*" or pneumonia* or SARS or "severe acute respiratory syndrome")) or (pneumonia adj3 (coronavirus* or "corona virus*" or betacoronavirus* or "beta coronavirus*" or "beta corona virus*" or SARS or "severe acute respiratory syndrome")) or 2019ncov or covid or covid19 or covid2019 or ncov or sarscov2).kf,kw,ti. or (coronavirus* or "corona virus*" or betacoronavirus* or "beta coronavirus*" or "beta corona virus*" or pneumonia* or SARS or "severe acute respiratory syndrome").ti. or ("2019 corona virus" or "2019 coronavirus" or "2019 ncov" or "corona virus 19" or "corona virus 2019" or "corona virus disease 19" or "corona virus disease 2019" or "corona virus epidemic*" or "corona virus outbreak*" or "corona virus pandemic*" or "corona virus response" or "coronavirus 19" or "coronavirus 2019" or "coronavirus disease 19" or "coronavirus disease 2019" or "coronavirus epidemic*" or "coronavirus outbreak*" or "coronavirus pandemic*" or "coronavirus response" or "covid 19" or "covid 2019" or "new corona virus" or "new coronavirus" or "novel corona virus" or "novel coronavirus" or "novel human coronavirus" or "sars coronavirus 2" or "sars cov 2" or "sars cov2" or "sars like coronavirus" or "severe acute respiratory syndrome corona virus 2" or "severe acute respiratory syndrome coronavirus 2" or "severe specific contagious pneumonia" or	251821

	"wuhan corona virus" or "wuhan coronavirus" or ((pandemic* or novel or wuhan) adj3 (coronavirus* or "corona virus*" or betacoronavirus* or "beta coronavirus*" or "beta corona virus*" or pneumonia* or SARS or "severe acute respiratory syndrome")) or (pneumonia adj3 (coronavirus* or "corona virus*" or betacoronavirus* or "beta coronavirus*" or "beta corona virus*" or SARS or "severe acute respiratory syndrome")) or 2019ncov or covid or covid19 or covid2019 or ncov or sarscov2).ab. /freq=2	
3	Immunization Programs/ or Immunization Schedule/ or Immunization, Secondary/ or Immunization, Passive/ or Immunization/ or Immunogenicity, Vaccine/ or Immunotherapy, Active/ or Mass Vaccination/ or Vaccination Coverage/ or Vaccination/ or Vaccines, Attenuated/ or Vaccines, Combined/ or Vaccines, Synthetic/ or Vaccines/ or Viral Vaccines/ or COVID-19 Vaccines/ or vaccine*.rn. or (vaccin* or immunis* or immuniz* or innoculat* or inoculat* or jab* or shot* or injection* or nonimmunis* or nonimmuniz* or postvaccin* or postimmunis* or postimmuniz* or reimmunis* or reimmuniz* or revaccinat* or unimmunis* or unimmuniz* or ((one or single two or double or first or second or partial* or full) adj2 (dose* or injection* or injected or injecting)) or single-dose* or double-dose* or BNT162* or Comirnaty or CoronaVac or Covishield or Tozinameran or "mrna-1273*" or ChAdOx1* or AZD1222 or "Ad26.COV2.S" or "JNJ-78436735" or Novavax or NVX-CoV2373 or Ad5-nCoV or aAPC or "lentiviral minigene" or vaxzevria).ti,ab,kf,kw.	1276813
4	2 and 3	25640
5	Adaptive Immunity/ or Antibodies, Neutralizing/ or Antibodies, Viral/ or Antibody Affinity/ or Antibody Formation/ or Antibody Specificity/ or Antibodies, Monoclonal/ or Binding Sites, Antibody/ or exp Antibodies/ or exp Antigen-Antibody Reactions/ or Antibody-Dependent Enhancement/ or Antibody-Dependent Cell Cytotoxicity/ or Lymphocytes/ or exp B-Lymphocytes/ or B-Lymphocyte subsets/ or Lymphocyte Activation/ or Complement Activation/ or Th2 Cells/ or Immune System Phenomena/ or Immunity/ or Immunity, Active/ or Immunity, Humoral/ or Immunity, Mucosal/ or Immunologic Factors/ or Immunologic Surveillance/ or Immunity, Innate/ or Immunity, Heterologous/ or Immunity, Maternally-Acquired/ or Immune Sera/ or exp Immunoglobulins/ or Immunoglobulin M/ or Immunoglobulin G/ or exp Immunomodulation/ or Immunologic Memory/ or Models, Immunological/ or Cross Protection/im or Cross Reactions/im or Reinfection/im or Recurrence/im or "Severity of Illness Index"/im or Viral Load/im or Convalescence/im or Seroconversion/ or Seroepidemiologic Studies/ or Neutralization Tests/ or Enzyme-Linked Immunosorbent Assay/ or Protein Domains/im or Receptors, Virus/im or Spike Glycoprotein, Coronavirus/im or Coronavirus Nucleocapsid Proteins/im or *COVID-19 Vaccines/im or *COVID-19/im or *SARS-CoV-2/im or (antibody or antibodies or antibody-positiv* or antibody-negativ* or "complement activation" or ((humoral or humoural or adaptive or antibody-mediated) adj3 (respon* or immun*)) or immunity	2782702

	or "immune system*" or "immune marker*" or ((immun* or sero*) adj3 respon*) or immunolog* or immunomodulat* or B-lymphocyte* or "B-cell*").ti,ab,kf,kw. or (immun* or immunoglobulin* or IgA or IgD or IgE or IgG or IgM or sero* or sera*).ti,kf,kw.	
6	("correlat* of protection" or "correlat* with protection" or "immune correlat*" or "correlat* of immunity" or "correlat* of neutraliz*" or "correlat* of neutralis*" or "measur* of protection" or "correlat* of infectivity" or "protective correlat*" or ((protective or protection) adj5 (level* or threshold* or marker* or limit* or amount* or mean* or baseline or quantif*)) or ((correlat* or marker* or threshold* or indicat* or measur*) adj5 (immun* or protect*)) or ((antibody or antibodies or titer* or titre*) adj3 (correlat* or threshold*)) or (protect* adj5 (immunity or antibod* or titer* or titre*)) or ((neutraliz* or neutralis*) adj2 (level* or amount* or limit* or mean* or baseline or quantif* or titer* or titre*)) or (predict* adj3 (immun* or protect*)) or "protective immunity" or "surrogate endpoint*").ti,ab,kf,kw. or (protection or protective or protected or ((antibody or antibodies) adj2 (status or level*))).ti.	370204
7	(1 or 4) and 5 and 6	2048
8	2 and 5 and 6	3863
9	7 or 8	3869
10	limit 9 to yr="2019 -Current"	2589
11	limit 10 to (english or french)	2559
12	Reinfection/ or Recurrence/ or Follow-Up Studies/ or ((Convalescence/ or "Severity of Illness Index"/ or Viral Load/ or Asymptomatic Infections/) and (re-infect* or reinfect*).ti,kf,kw.)	827685
13	(((previous* or prior or past or post or postcovid* or post-covid* or postsars* or post-sars* or after or following or recover*) adj2 infect*) or re-infect* or reinfect* or "after first infection" or "after infection").ti,ab,kf,kw. or (convalescence or convalescent).ti,kf,kw.	135528
14	12 or 13	955646
15	Treatment Failure/ or ((vaccin* or immunis* or immuniz*) adj3 fail*).ab,kf,kw,ti.	39442
16	(((breakthrough* or "break through*") adj5 (vaccin* or infection*)) or "breakthrough rate*").ab,kf,kw,ti.	1398
17	(("second dose" or "two doses" or "fully vaccinated") adj5 (infected or infection* or contracted or acquired or acquisition or caught or got* or transmi* or spread* or outbreak* or cluster* or communicab* or contagious* or epidemic* or occurrence or case or cases)).ab,kf,kw,ti.	378
18	((postvaccin* or postimmuniz* or postimmunis* or "post-vaccin*" or "post-immuniz*" or "post-immunis*") adj5 (infected or infection* or contracted or acquired or acquisition or caught or got* or transmi* or spread* or outbreak* or cluster* or communicab* or contagious* or epidemic* or occurrence or case or cases)).ab,kf,kw,ti.	618

19	(((receiv* or recipient* or complet* or already or post or previously or after or following or "subsequent to" or since or had or despite or "in spite of") adj5 (immunis* or immuniz* or inoculat* or innoculat* or vaccin* or revaccinat* or reimmuniz* or reimmunis* or dose or doses) adj5 (infected or infection* or contracted or acquired or acquisition or caught or got* or transmi* or spread* or outbreak* or cluster* or communicab* or contagious* or epidemic* or occurrence or case or cases)) or ((infected or infection* or contracted or acquired or acquisition or caught or got* or transmi* or spread* or outbreak* or cluster* or communicab* or contagious* or epidemic* or occurrence or case or cases) adj5 (receiv* or recipient* or complet* or already or post or previously or after or following or "subsequent to" or since or had or despite or "in spite of") adj5 (immunis* or immuniz* or inoculat* or innoculat* or vaccin* or revaccinat* or reimmuniz* or reimmunis* or dose or doses))).ab,kf,kw,ti.	19047
20	((vaccinated or immunized or immunised) adj5 (infected or infection* or contracted or acquired or acquisition or caught or got* or transmi* or spread* or outbreak* or cluster* or communicab* or contagious* or epidemic* or occurrence or case or cases)).ab,kf,kw,ti.	8545
21	((vaccinated or immunized or immunised) adj10 (person or persons or people or man or men or woman or women or adult or adults or patient or patients or individual or individuals or responder or responders or practitioner* or staff or personnel or worker* or employee* or provider* or technician* or clinician* or doctor* or nurse or nurses or paramedic* or physician* or hospitalist* or pharmacist*) adj10 (infected or infection* or contracted or acquired or acquisition or caught or got* or transmi* or spread* or outbreak* or cluster* or communicab* or contagious* or epidemic* or occurrence or case or cases)).ab,kf,kw,ti.	2764
22	((escape or escapes or escaped or escaping or evade or evades or evaded or evading or evasion) adj5 ("Ad26.COV2.S" or AZD1222 or BNT162* or ChAdOx1* or Comirnaty or immunis* or immuniz* or "JNJ-78436735" or "mrna-1273*" or Novavax or NVX-CoV2373 or PittCoVacc* or postvaccin* or postimmunis* or postimmuniz* or reimmunis* or reimmuniz* or revacciat* or tozinameran or vaccin*)).ab,kf,kw,ti.	992
23	15 or 16 or 17 or 18 or 19 or 20 or 21 or 22	67744
24	(wane or wanes or waned or waning).kf,kw,ti.	559
25	((decay* or declin* or decreas* or impair* or duration or fail* or fall* or "go* down" or lack or lacks or lacked or lacking or lose or loses or losing or loss or lost or low or lower* or lesser* or poor or poorer* or "adversely affect*" or reduc*) adj5 (protect* or effectiv* or immunity or "immune respon*" or antibod* or sero*)).kf,kw,ti. and (infected or infection* or contracted or acquired or acquisition or caught or got* or transmi* or spread* or outbreak* or cluster* or communicab* or contagious* or epidemic* or occurrence or case or cases).ab,kf,kw,ti.	6132

26	((decay* or declin* or decreas* or impair* or duration or fail* or fall* or "go* down" or lack or lacks or lacked or lacking or lose or loses or losing or loss or lost or low or lower* or lesser* or poor or poorer* or "adversely affect*" or reduc*) adj5 (protect* or effectiv* or immunity or "immune respon*" or antibod* or sero*)).ab,kf,kw,ti. and (infected or infection* or contracted or acquired or acquisition or caught or got* or transmi* or spread* or outbreak* or cluster* or communicab* or contagious* or epidemic* or occurrence or case or cases).kf,kw,ti.	29587
27	24 or 25 or 26	34166
28	(1 or 4) and (14 or 23 or 27) and (5 and 6)	595
29	2 and (14 or 27) and (5 and 6)	876
30	28 or 29	993
31	limit 30 to yr="2019 -Current"	739
32	limit 31 to (english or french)	735
33	11 not 32	1824

09/03/2021

COVID-19 antibody correlates of protection: Supplementary Databases

Request prepared by: Library Services
Contact information: library@oahpp.ca

Search results reporting

DATABASES SEARCHED

Database	Date searched	Records	Duplicates removed by database	Remaining
EMBASE (I)	09/03/2021	1595	0	1595
EMBASE (II)	09/03/2021	473	0	473
GLOBAL HEALTH (I)	09/03/2021	803	0	803
GLOBAL HEALTH (II)	09/03/2021	306	0	306
BIOSIS Previews (I)	09/03/2021	819	0	819
BIOSIS Previews (II)	09/03/2021	359	0	359
SCOPUS	09/03/2021	692	0	692

RESULTS TOTALS

Records source	Records
Records identified through database searching	5047
Duplicates removed by database	0
Duplicates removed by bibliographic management software	4,159
Total records after duplicates removed<br>*(Search I combined: 766 records)*<br>*(Search II combined: 122 records)*	888

Search strategies

EMBASE

Embase <1974 to 2021 Week 34>

#	Searches	Results
1	SARS-CoV-2 vaccine/ or ("Ad5-nCoV vaccine" or "BNT162 vaccine" or "ChAdOx1 COVID-19 vaccine" or "Covid-19 aAPC vaccine" or "lentiviral minigene vaccine of COVID-19 coronavirus" or "mRNA-1273 vaccine" or "PittCoVacc" or "recombinant SARS-CoV-2 vaccine NVX-cov2373" or "SARS-CoV-2 inactivated vaccines").rn. or (BNT162* or Comirnaty or CoronaVac or Covishield or Tozinameran or "mrna-1273*" or ChAdOx1* or AZD1222 or "Ad26.COV2.S" or "JNJ-78436735" or Novavax or NVX-CoV2373 or PittCoVacc* or Ad5-nCoV or aAPC or "lentiviral minigene" or vaxzevria).ti,kw.	5424
2	coronavirus disease 2019/ or Severe acute respiratory syndrome coronavirus 2/ or (pandemic/ and Coronavirus infection/) or ("2019 corona virus" or "2019 coronavirus" or "2019 ncov" or "corona virus 19" or "corona virus 2019" or "corona virus disease 19" or "corona virus disease 2019" or "corona virus epidemic*" or "corona virus outbreak*" or "corona virus pandemic*" or "corona virus response" or "coronavirus 19" or "coronavirus 2019" or "coronavirus disease 19" or "coronavirus disease 2019" or "coronavirus epidemic*" or "coronavirus outbreak*" or "coronavirus pandemic*" or "coronavirus response" or "covid 19" or "covid 2019" or "new corona virus" or "new coronavirus" or "novel corona virus" or "novel coronavirus" or "novel human coronavirus" or "sars coronavirus 2" or "sars cov 2" or "sars cov2" or "sars like coronavirus" or "severe acute respiratory syndrome corona virus 2" or "severe acute respiratory syndrome coronavirus 2" or "severe specific contagious pneumonia" or "wuhan corona virus" or "wuhan coronavirus" or ((pandemic* or novel or wuhan) adj3 (coronavirus* or "corona virus*" or betacoronavirus* or "beta coronavirus*" or "beta corona virus*" or pneumonia* or SARS or "severe acute respiratory syndrome")) or (pneumonia adj3 (coronavirus* or "corona virus*" or betacoronavirus* or "beta coronavirus*" or "beta corona virus*" or SARS or "severe acute respiratory syndrome")) or 2019ncov or covid or covid19 or covid2019 or ncov or sarscov2).kw,ti. or ("SARS-Cov-2 variant*" or VUI-202012-01 or "VOC-202012/01" or "B.1.1.7" or B117 or "B.1.351" or B1351 or "B.1.617" or B1617 or "P.1" or P1 or coronavirus* or "corona virus*" or betacoronavirus* or "beta coronavirus*" or "beta corona virus*" or pneumonia* or SARS or "severe acute respiratory syndrome").ti. or	279851

	((covid* or sars* or variant*) and (alpha or beta or gamma or delta or "variant* of concern" or "VOC")).ti.	
3	immunization/ or secondary immunization/ or vaccine immunogenicity/ or active immunotherapy/ or mass immunization/ or vaccination/ or vaccination coverage/ or vaccination refusal/ or live vaccine/ or vaccine/ or recombinant vaccine/ or virus vaccine/ or SARS-CoV-2 vaccine/ or (vaccin* or immunis* or immuniz* or innoculat* or inoculat* or jab* or shot* or injection* or nonimmunis* or nonimmuniz* or postvaccin* or postimmunis* or postimmuniz* or reimmunis* or reimmuniz* or revaccinat* or unimmunis* or unimmuniz* or ((one or single two or double or first or second or partial* or full) adj2 (dose* or injection* or injected or injecting)) or single-dose* or double-dose* or BNT162* or Comirnaty or CoronaVac or Covishield or Tozinameran or "mrna-1273*" or ChAdOx1* or AZD1222 or "Ad26.COV2.S" or "JNJ-78436735" or Novavax or NVX-CoV2373 or Ad5-nCoV or aAPC or "lentiviral minigene" or vaxzevria).ti,ab,kw.	1572516
4	2 and 3	28195
5	adaptive immunity/ or neutralizing antibody/ or virus antibody/ or antibody affinity/ or antibody production/ or antibody specificity/ or monoclonal antibody/ or antibody combining site/ or exp antibody/ or exp antigen antibody reaction/ or antibody dependent enhancement/ or antibody dependent cellular cytotoxicity/ or antibody response/ or lymphocyte/ or exp B lymphocyte/ or B lymphocyte subpopulation/ or lymphocyte activation/ or complement activation/ or Th2 cell/ or immunity/ or active immunization/ or humoral immunity/ or mucosal immunity/ or immunologic factor/ or immunosurveillance/ or innate immunity/ or heterologous immunity/ or passive immunization/ or antiserum/ or exp immunoglobulin/ or immunoglobulin G/ or immunological memory/ or biological model/ or cross protection/ or cross reaction/ or reinfection/ or recurrent disease/ or "severity of illness index"/ or virus load/ or convalescence/ or seroconversion/ or seroepidemiology/ or serodiagnosis/ or enzyme linked immunosorbent assay/ or protein domain/ or virus receptor/ or coronavirus spike glycoprotein/ or nucleocapsid protein/ or (antibody or antibodies or antibody-positiv* or antibody-negativ* or "complement activation" or ((humoral or humoural or adaptive or antibody-mediated) adj3 (respon* or immun*)) or immunity or "immune system*" or "immune marker*" or ((immun* or sero*) adj3 respon*) or immunolog* or immunomodulat* or B-lymphocyte* or "B-cell*").ti,kw. or (antibody or antibodies or antibody-positiv* or antibody-negativ* or "complement activation" or ((humoral or humoural or adaptive or antibody-mediated) adj3 (respon* or immun*)) or immunity or "immune system*" or "immune marker*" or ((immun* or sero*) adj3 respon*) or immunolog* or immunomodulat* or B-lymphocyte* or "B-cell*").ab. /freq=2 or (immun* or immunoglobulin* or IgA or IgD or IgE or IgG or IgM or sero* or sera*).ti,kw.	3948248

6	("correlat* of protection" or "correlat* with protection" or "immune correlat*" or "correlat* of immunity" or "correlat* of neutraliz*" or "correlat* of neutralis*" or "measur* of protection" or "correlat* of infectivity" or "protective correlat*" or ((protective or protection) adj5 (level* or threshold* or marker* or limit* or amount* or mean* or baseline or quantif*)) or ((correlat* or marker* or threshold* or indicat* or measur*) adj5 (immun* or protect*)) or ((antibody or antibodies or titer* or titre*) adj3 (correlat* or threshold*)) or (protect* adj5 (immunity or antibod* or titer* or titre*)) or ((neutraliz* or neutralis*) adj2 (level* or amount* or limit* or mean* or baseline or quantif* or titer* or titre*)) or (predict* adj3 (immun* or protect*)) or "protective immunity" or "surrogate endpoint*").ti,ab,kw. or (protection or protective or protected or ((antibody or antibodies) adj2 (status or level*))).ti.	474113
7	(1 or 4) and 5 and 6	1908
8	2 and 5 and 6	4025
9	7 or 8	4038
10	limit 9 to conference abstract	412
11	9 not 10	3626
12	11 not (editorial or letter or note).pt.	3467
13	limit 12 to yr="2019 -Current"	2123
14	limit 13 to (english or french)	2068
15	reinfection/ or recurrent disease/ or ((follow up/ or convalescence/ or "severity of illness index"/ or virus load/ or asymptomatic infection/) and (re-infect* or reinfect*).ti,kw.)	199957
16	(((previous* or prior or past or post or postcovid* or post-covid* or postsars* or post-sars* or after or following or recover* or convalescen* or "after infection") adj2 infect*) or re-infect* or reinfect* or "after first infection").ti,ab,kw.	167755
17	15 or 16	358275
18	treatment failure/ or ((vaccin* or immunis* or immuniz*) adj3 fail*).ab,kw,ti.	142466
19	(((breakthrough* or "break through*") adj5 (vaccin* or infection*)) or "breakthrough rate*").ab,kw,ti.	1940
20	(("second dose" or "two doses" or "fully vaccinated") adj5 (infected or infection* or contracted or acquired or acquisition or caught or got* or transmi* or spread* or outbreak* or cluster* or communicab* or contagious* or epidemic* or occurrence or case or cases)).ab,kw,ti.	476
21	((postvaccin* or postimmuniz* or postimmunis* or "post-vaccin*" or "post-immuniz*" or "post-immunis*") adj5 (infected or infection* or contracted or acquired or acquisition or caught or got* or transmi* or spread* or outbreak* or cluster* or communicab* or contagious* or epidemic* or occurrence or case or cases)).ab,kw,ti.	716
22	(((receiv* or recipient* or complet* or already or post or previously or after or following or "subsequent to" or since or had or despite or "in spite of") adj5 (immunis* or immuniz* or inoculat* or innoculat* or vaccin* or revaccinat* or	23864

	reimmuniz* or reimmunis* or dose or doses) adj5 (infected or infection* or contracted or acquired or acquisition or caught or got* or transmi* or spread* or outbreak* or cluster* or communicab* or contagious* or epidemic* or occurrence or case or cases)) or ((infected or infection* or contracted or acquired or acquisition or caught or got* or transmi* or spread* or outbreak* or cluster* or communicab* or contagious* or epidemic* or occurrence or case or cases) adj5 (receiv* or recipient* or complet* or already or post or previously or after or following or "subsequent to" or since or had or despite or "in spite of") adj5 (immunis* or immuniz* or inoculat* or innoculat* or vaccin* or revaccinat* or reimmuniz* or reimmunis* or dose or doses))).ab,kw,ti.	
23	((vaccinated or immunized or immunised) adj5 (infected or infection* or contracted or acquired or acquisition or caught or got* or transmi* or spread* or outbreak* or cluster* or communicab* or contagious* or epidemic* or occurrence or case or cases)).ab,kw,ti.	9423
24	((vaccinated or immunized or immunised) adj10 (person or persons or people or man or men or woman or women or adult or adults or patient or patients or individual or individuals or responder or responders or practitioner* or staff or personnel or worker* or employee* or provider* or technician* or clinician* or doctor* or nurse or nurses or paramedic* or physician* or hospitalist* or pharmacist*) adj10 (infected or infection* or contracted or acquired or acquisition or caught or got* or transmi* or spread* or outbreak* or cluster* or communicab* or contagious* or epidemic* or occurrence or case or cases)).ab,kw,ti.	3485
25	((escape or escapes or escaped or escaping or evade or evades or evaded or evading or evasion) adj5 ("Ad26.COV2.S" or AZD1222 or BNT162* or ChAdOx1* or Comirnaty or immunis* or immuniz* or "JNJ-78436735" or "mrna-1273*" or Novavax or NVX-CoV2373 or PittCoVacc* or postvaccin* or postimmunis* or postimmuniz* or reimmunis* or reimmuniz* or revacciat* or tozinameran or vaccin*)).ab,kw,ti.	1175
26	18 or 19 or 20 or 21 or 22 or 23 or 24 or 25	176896
27	(wane or wanes or waned or waning).kw,ti.	646
28	((decay* or declin* or decreas* or impair* or duration or fail* or fall* or "go* down" or lack or lacks or lacked or lacking or lose or loses or losing or loss or lost or low or lower* or lesser* or poor or poorer* or "adversely affect*" or reduc*) adj5 (protect* or effectiv* or immunity or "immune respon*" or antibod* or sero*)).kw,ti. and (infected or infection* or contracted or acquired or acquisition or caught or got* or transmi* or spread* or outbreak* or cluster* or communicab* or contagious* or epidemic* or occurrence or case or cases).ab,kw,ti.	8059
29	((decay* or declin* or decreas* or impair* or duration or fail* or fall* or "go* down" or lack or lacks or lacked or lacking or lose or loses or losing or loss or lost or low or lower* or lesser* or poor or poorer* or "adversely affect*" or reduc*) adj5 (protect* or effectiv* or immunity or "immune respon*" or antibod* or sero*)).ab,kw,ti. and	38103

	(infected or infection* or contracted or acquired or acquisition or caught or got* or transmi* or spread* or outbreak* or cluster* or communicab* or contagious* or epidemic* or occurrence or case or cases).kw,ti.	
30	27 or 28 or 29	44270
31	(1 or 4) and (17 or 26 or 30) and (5 and 6)	477
32	2 and (17 or 30) and (5 and 6)	731
33	31 or 32	840
34	limit 33 to conference abstract	100
35	33 not 34	740
36	35 not (editorial or letter or note).pt.	720
37	limit 36 to yr="2019 -Current"	482
38	limit 37 to (english or french)	473
39	14 not 38	1595

GLOBAL HEALTH

Global Health <1973 to 2021 Week 35>

#	Searches	Results
1	((vaccines/ or immunization/ or vaccination/ or synthetic vaccines/ or combined vaccines/ or vaccine development/ or candidate vaccines/ or messenger RNA/) and (ncov or BNT162* or "mrna-1273").ti,ab,hw.) or (BNT162* or Comirnaty or CoronaVac or Covishield or Tozinameran or "mrna-1273*" or ChAdOx1* or AZD1222 or "Ad26.COV2.S" or "JNJ-78436735" or Novavax or NVX-CoV2373 or Ad5-nCoV or aAPC or "lentiviral minigene" or vaxzevria).ti,ab,hw.	590
2	("Severe acute respiratory syndrome coronavirus 2" or "coronavirus disease 2019" or "COVID-19" or "SARS-CoV-2").ti,hw,id. or ("2019 corona virus" or "2019 coronavirus" or "2019 ncov" or "corona virus 19" or "corona virus 2019" or "corona virus disease 19" or "corona virus disease 2019" or "corona virus epidemic*" or "corona virus outbreak*" or "corona virus pandemic*" or "corona virus response" or "coronavirus 19" or "coronavirus 2019" or "coronavirus disease 19" or "coronavirus disease 2019" or "coronavirus epidemic*" or "coronavirus outbreak*" or "coronavirus pandemic*" or "coronavirus response" or "covid 19" or "covid 2019" or "new corona virus" or "new coronavirus" or "novel corona virus" or "novel coronavirus" or "novel human coronavirus" or "sars coronavirus 2" or "sars cov 2" or "sars cov2" or "sars like coronavirus" or "severe acute respiratory syndrome corona virus 2" or "severe acute respiratory syndrome coronavirus 2" or "severe specific contagious pneumonia" or "wuhan corona virus" or "wuhan coronavirus" or ((pandemic* or novel or wuhan) adj3 (coronavirus* or "corona virus*" or betacoronavirus* or "beta coronavirus*" or "beta corona virus*" or pneumonia* or SARS or "severe acute respiratory syndrome")) or (pneumonia adj3 (coronavirus* or "corona virus*" or betacoronavirus* or "beta	73545

	coronavirus*" or "beta corona virus*" or SARS or "severe acute respiratory syndrome")) or 2019ncov or covid or covid19 or covid2019 or ncov or sarscov2).ti,hw. or ("SARS-Cov-2 variant*" or VUI-202012-01 or "VOC-202012/01" or "B.1.1.7" or B117 or "B.1.351" or B1351 or "B.1.617" or B1617 or "P.1" or P1 or coronavirus* or "corona virus*" or betacoronavirus* or "beta coronavirus*" or "beta corona virus*" or pneumonia* or SARS or "severe acute respiratory syndrome").ti. or ((covid* or sars* or variant*) and (alpha or beta or gamma or delta or "variant* of concern" or "VOC")).ti. or ("2019 corona virus" or "2019 coronavirus" or "2019 ncov" or "corona virus 19" or "corona virus 2019" or "corona virus disease 19" or "corona virus disease 2019" or "corona virus epidemic*" or "corona virus outbreak*" or "corona virus pandemic*" or "corona virus response" or "coronavirus 19" or "coronavirus 2019" or "coronavirus disease 19" or "coronavirus disease 2019" or "coronavirus epidemic*" or "coronavirus outbreak*" or "coronavirus pandemic*" or "coronavirus response" or "covid 19" or "covid 2019" or "new corona virus" or "new coronavirus" or "novel corona virus" or "novel coronavirus" or "novel human coronavirus" or "sars coronavirus 2" or "sars cov 2" or "sars cov2" or "sars like coronavirus" or "severe acute respiratory syndrome corona virus 2" or "severe acute respiratory syndrome coronavirus 2" or "severe specific contagious pneumonia" or "wuhan corona virus" or "wuhan coronavirus" or ((pandemic* or novel or wuhan) adj3 (coronavirus* or "corona virus*" or betacoronavirus* or "beta coronavirus*" or "beta corona virus*" or pneumonia* or SARS or "severe acute respiratory syndrome")) or (pneumonia adj3 (coronavirus* or "corona virus*" or betacoronavirus* or "beta coronavirus*" or "beta corona virus*" or SARS or "severe acute respiratory syndrome")) or 2019ncov or covid or covid19 or covid2019 or ncov or sarscov2).ab. /freq=2	
3	vaccines/ or immunization programmes/ or immunization/ or vaccination/ or passive immunization/ or synthetic vaccines/ or combined vaccines/ or vaccine development/ or candidate vaccines/ or (vaccin* or immunis* or immuniz* or innoculat* or inoculat* or jab* or shot* or injection* or nonimmunis* or nonimmuniz* or postvaccin* or postimmunis* or postimmuniz* or reimmunis* or reimmuniz* or revaccinat* or unimmunis* or unimmuniz* or ((one or single two or double or first or second or partial* or full) adj2 (dose* or injection* or injected or injecting)) or single-dose* or double-dose* or BNT162* or Comirnaty or CoronaVac or Covishield or Tozinameran or "mrna-1273*" or ChAdOx1* or AZD1222 or "Ad26.COV2.S" or "JNJ-78436735" or Novavax or NVX-CoV2373 or Ad5-nCoV or aAPC or "lentiviral minigene" or vaxzevria).ti,ab,hw.	275347
4	2 and 3	8485
5	neutralizing antibodies/ or monoclonal antibodies/ or exp antibodies/ or antibody formation/ or antibody testing/ or antigen antibody reactions/ or humoral immunity/ or immune serum/ or immunity/ or immune response/ or immune system/ or cross immunity/ or cross reaction/ or passive immunity/ or immune competence/ or	669065

	immunological factors/ or immunology/ or antibody dependent cellular cytotoxicity/ or lymphocytes/ or B lymphocytes/ or lymphocyte transformation/ or complement activation/ or Th2 lymphocytes/ or exp immunoglobulins/ or reinfection/ or viral load/ or seroconversion/ or seroprevalence/ or neutralization tests/ or virus neutralization/ or ELISA/ or (antibody or antibodies or antibody-positiv* or antibody-negativ* or "complement activation" or ((humoral or humoural or adaptive or antibody-mediated) adj3 (respon* or immun*)) or immunity or "immune system*" or "immune marker*" or ((immun* or sero*) adj3 respon*) or immunolog* or immunomodulat* or B-lymphocyte* or "B-cell*").ti,ab,hw. or (immun* or immunoglobulin* or IgA or IgD or IgE or IgG or IgM or sero* or sera*).ti,hw.	
6	("correlat* of protection" or "correlat* with protection" or "immune correlat*" or "correlat* of immunity" or "correlat* of neutraliz*" or "correlat* of neutralis*" or "measur* of protection" or "correlat* of infectivity" or "protective correlat*" or ((protective or protection) adj5 (level* or threshold* or marker* or limit* or amount* or mean* or baseline or quantif*)) or ((correlat* or marker* or threshold* or indicat* or measur*) adj5 (immun* or protect*)) or ((antibody or antibodies or titer* or titre*) adj3 (correlat* or threshold*)) or (protect* adj5 (immunity or antibod* or titer* or titre*)) or ((neutraliz* or neutralis*) adj2 (level* or amount* or limit* or mean* or baseline or quantif* or titer* or titre*)) or (predict* adj3 (immun* or protect*)) or "protective immunity" or "surrogate endpoint*").ti,ab,hw. or (protection or protective or protected or ((antibody or antibodies) adj2 (status or level*))).ti.	84772
7	5 and 6	47062
8	(1 or 4) and 7	679
9	2 and 7	1505
10	8 or 9	1518
11	limit 10 to yr="2019 -Current"	1135
12	limit 11 to (english or french)	1109
13	reinfection/ or ((viral load/ or asymptomatic infections/) and (re-infect* or reinfect*).ti,hw.)	2358
14	(((previous* or prior or past or post or postcovid* or post-covid* or postsars* or post-sars* or after or following or recover* or convalescen* or "after infection") adj3 infect*) or re-infect* or reinfect* or "after first infection").ti,ab,hw.	59349
15	13 or 14	59349
16	treatment failure/ or ((vaccin* or immunis* or immuniz*) adj3 fail*).ti,ab,hw.	10182
17	(((breakthrough* or "break through*") adj5 (vaccin* or infection*)) or "breakthrough rate*").ti,ab,hw.	711
18	(("second dose" or "two doses" or "fully vaccinated") adj5 (infected or infection* or contracted or acquired or acquisition or caught or got* or transmi* or spread* or outbreak* or cluster* or communicab* or contagious* or epidemic* or occurrence or case or cases)).ti,ab,hw.	189

19	((postvaccin* or postimmuniz* or postimmunis* or "post-vaccin*" or "post-immuniz*" or "post-immunis*") adj5 (infected or infection* or contracted or acquired or acquisition or caught or got* or transmi* or spread* or outbreak* or cluster* or communicab* or contagious* or epidemic* or occurrence or case or cases)).ti,ab,hw.	253
20	(((receiv* or recipient* or complet* or already or post or previously or after or following or "subsequent to" or since or had or despite or "in spite of") adj5 (immunis* or immuniz* or inoculat* or innoculat* or vaccin* or revaccinat* or reimmuniz* or reimmunis* or dose or doses) adj5 (infected or infection* or contracted or acquired or acquisition or caught or got* or transmi* or spread* or outbreak* or cluster* or communicab* or contagious* or epidemic* or occurrence or case or cases)) or ((infected or infection* or contracted or acquired or acquisition or caught or got* or transmi* or spread* or outbreak* or cluster* or communicab* or contagious* or epidemic* or occurrence or case or cases) adj5 (receiv* or recipient* or complet* or already or post or previously or after or following or "subsequent to" or since or had or despite or "in spite of") adj5 (immunis* or immuniz* or inoculat* or innoculat* or vaccin* or revaccinat* or reimmuniz* or reimmunis* or dose or doses))).ti,ab,hw.	7993
21	((vaccinated or immunized or immunised) adj5 (infected or infection* or contracted or acquired or acquisition or caught or got* or transmi* or spread* or outbreak* or cluster* or communicab* or contagious* or epidemic* or occurrence or case or cases)).ti,ab,hw.	3928
22	((vaccinated or immunized or immunised) adj10 (person or persons or people or man or men or woman or women or adult or adults or patient or patients or individual or individuals or responder or responders or practitioner* or staff or personnel or worker* or employee* or provider* or technician* or clinician* or doctor* or nurse or nurses or paramedic* or physician* or hospitalist* or pharmacist*) adj10 (infected or infection* or contracted or acquired or acquisition or caught or got* or transmi* or spread* or outbreak* or cluster* or communicab* or contagious* or epidemic* or occurrence or case or cases)).ti,ab,hw.	1646
23	((escape or escapes or escaped or escaping or evade or evades or evaded or evading or evasion) adj5 ("Ad26.COV2.S" or AZD1222 or BNT162* or ChAdOx1* or Comirnaty or immunis* or immuniz* or "JNJ-78436735" or "mrna-1273*" or Novavax or NVX-CoV2373 or PittCoVacc* or postvaccin* or postimmunis* or postimmuniz* or reimmunis* or reimmuniz* or revacciat* or tozinameran or vaccin*)).ti,ab,hw.	399
24	16 or 17 or 18 or 19 or 20 or 21 or 22 or 23	22285
25	(wane or wanes or waned or waning).ti,hw.	119
26	((decay* or declin* or decreas* or impair* or duration or fail* or fall* or "go* down" or lack or lacks or lacked or lacking or lose or loses or losing or loss or lost or low or lower* or lesser* or poor or poorer* or "adversely affect*" or reduc*) adj5 (protect* or effectiv* or immunity or "immune respon*" or antibod* or sero*)).ti,hw. and (infected or infection* or contracted or acquired or acquisition or caught or got* or transmi* or	2831

	spread* or outbreak* or cluster* or communicab* or contagious* or epidemic* or occurrence or case or cases).ti,ab,hw.	
27	((decay* or declin* or decreas* or impair* or duration or fail* or fall* or "go* down" or lack or lacks or lacked or lacking or lose or loses or losing or loss or lost or low or lower* or lesser* or poor or poorer* or "adversely affect*" or reduc*) adj5 (protect* or effectiv* or immunity or "immune respon*" or antibod* or sero*)).ab,ti,hw. and (infected or infection* or contracted or acquired or acquisition or caught or got* or transmi* or spread* or outbreak* or cluster* or communicab* or contagious* or epidemic* or occurrence or case or cases).hw,ti.	34252
28	25 or 26 or 27	34783
29	(1 or 4) and (15 or 24 or 28) and (5 and 6)	226
30	2 and (15 or 28) and (5 and 6)	373
31	29 or 30	406
32	limit 31 to yr="2019 -Current"	313
33	limit 32 to (english or french)	306
34	12 not 33	803

BIOSIS

BIOSIS Previews <2021 Week 01 to 2021 Week 41>

	Searches	Results
1	(ncov or BNT162* or "mrna-1273" or "Ad5-nCoV vaccine" or "BNT162 vaccine" or "ChAdOx1 COVID-19 vaccine" or "Covid-19 aAPC vaccine" or "lentiviral minigene vaccine of COVID-19 coronavirus" or "mRNA-1273 vaccine" or "recombinant SARS-CoV-2 vaccine NVX-cov2373" or "SARS-CoV-2 inactivated vaccines").rn. or (BNT162* or Comirnaty or CoronaVac or Covishield or Tozinameran or "mrna-1273*" or ChAdOx1* or AZD1222 or "Ad26.COV2.S" or "JNJ-78436735" or Novavax or NVX-CoV2373 or Ad5-nCoV or aAPC or "lentiviral minigene" or vaxzevria).ti,tw.	400
2	("Severe acute respiratory syndrome coronavirus 2" or "coronavirus disease 2019" or "COVID-19" or "SARS-CoV-2" or ("2019 corona virus" or "2019 coronavirus" or "2019 ncov" or "corona virus 19" or "corona virus 2019" or "corona virus disease 19" or "corona virus disease 2019" or "corona virus epidemic*" or "corona virus outbreak*" or "corona virus pandemic*" or "corona virus response" or "coronavirus 19" or "coronavirus 2019" or "coronavirus disease 19" or "coronavirus disease 2019" or "coronavirus epidemic*" or "coronavirus outbreak*" or "coronavirus pandemic*" or "coronavirus response" or "covid 19" or "covid 2019" or "new corona virus" or "new coronavirus" or "novel corona virus" or "novel coronavirus" or "novel human coronavirus" or "sars coronavirus 2" or "sars cov 2" or "sars cov2" or "sars like coronavirus" or "severe acute respiratory syndrome corona virus 2" or "severe acute respiratory syndrome coronavirus 2" or "severe specific contagious pneumonia" or	41503

	"wuhan corona virus" or "wuhan coronavirus" or ((pandemic* or novel or wuhan) adj3 (coronavirus* or "corona virus*" or betacoronavirus* or "beta coronavirus*" or "beta corona virus*" or pneumonia* or SARS or "severe acute respiratory syndrome")) or (pneumonia adj3 (coronavirus* or "corona virus*" or betacoronavirus* or "beta coronavirus*" or "beta corona virus*" or SARS or "severe acute respiratory syndrome")) or 2019ncov or covid or covid19 or covid2019 or ncov or sarscov2)).ti,hw,mc. or ("SARS-Cov-2 variant*" or VUI-202012-01 or "VOC-202012/01" or "B.1.1.7" or B117 or "B.1.351" or B1351 or "B.1.617" or B1617 or "P.1" or P1 or coronavirus* or "corona virus*" or betacoronavirus* or "beta coronavirus*" or "beta corona virus*" or pneumonia* or SARS or "severe acute respiratory syndrome").ti. or ((covid* or sars* or variant*) and (alpha or beta or gamma or delta or "variant* of concern" or "VOC")).ti. or ("2019 corona virus" or "2019 coronavirus" or "2019 ncov" or "corona virus 19" or "corona virus 2019" or "corona virus disease 19" or "corona virus disease 2019" or "corona virus epidemic*" or "corona virus outbreak*" or "corona virus pandemic*" or "corona virus response" or "coronavirus 19" or "coronavirus 2019" or "coronavirus disease 19" or "coronavirus disease 2019" or "coronavirus epidemic*" or "coronavirus outbreak*" or "coronavirus pandemic*" or "coronavirus response" or "covid 19" or "covid 2019" or "new corona virus" or "new coronavirus" or "novel corona virus" or "novel coronavirus" or "novel human coronavirus" or "sars coronavirus 2" or "sars cov 2" or "sars cov2" or "sars like coronavirus" or "severe acute respiratory syndrome corona virus 2" or "severe acute respiratory syndrome coronavirus 2" or "severe specific contagious pneumonia" or "wuhan corona virus" or "wuhan coronavirus" or ((pandemic* or novel or wuhan) adj3 (coronavirus* or "corona virus*" or betacoronavirus* or "beta coronavirus*" or "beta corona virus*" or pneumonia* or SARS or "severe acute respiratory syndrome")) or (pneumonia adj3 (coronavirus* or "corona virus*" or betacoronavirus* or "beta coronavirus*" or "beta corona virus*" or SARS or "severe acute respiratory syndrome")) or 2019ncov or covid or covid19 or covid2019 or ncov or sarscov2).ab. /freq=2	
3	(vaccin* or immunis* or immuniz* or innoculat* or inoculat* or jab* or shot* or injection* or nonimmunis* or nonimmuniz* or postvaccin* or postimmunis* or postimmuniz* or reimmunis* or reimmuniz* or revaccinat* or unimmunis* or unimmuniz* or ((one or single two or double or first or second or partial* or full) adj2 (dose* or injection* or injected or injecting)) or single-dose* or double-dose* or BNT162* or Comirnaty or CoronaVac or Covishield or Tozinameran or "mrna-1273*" or ChAdOx1* or AZD1222 or "Ad26.COV2.S" or "JNJ-78436735" or Novavax or NVX-CoV2373 or Ad5-nCoV or aAPC or "lentiviral minigene" or vaxzevria).ti,ab,hw,tw,mc.	67637
4	2 and 3	6024
5	(antibody or antibodies or antibody-positiv* or antibody-negativ* or "complement activation" or ((humoral or humoural or adaptive or antibody-mediated) adj3 (respon* or immun*)) or immunity or "immune system*" or "immune marker*" or ((immun* or	288794

	sero*) adj3 respon*) or immunolog* or immunomodulat* or B-lymphocyte* or "B-cell*").ti,ab,hw,tw,mc. or (immun* or immunoglobulin* or IgA or IgD or IgE or IgG or IgM or sero* or sera*).ti,hw.	
6	("correlat* of protection" or "correlat* with protection" or "immune correlat*" or "correlat* of immunity" or "correlat* of neutraliz*" or "correlat* of neutralis*" or "measur* of protection" or "correlat* of infectivity" or "protective correlat*" or ((protective or protection) adj5 (level* or threshold* or marker* or limit* or amount* or mean* or baseline or quantif*)) or ((correlat* or marker* or threshold* or indicat* or measur*) adj5 (immun* or protect*)) or ((antibody or antibodies or titer* or titre*) adj3 (correlat* or threshold*)) or (protect* adj5 (immunity or antibod* or titer* or titre*)) or ((neutraliz* or neutralis*) adj2 (level* or amount* or limit* or mean* or baseline or quantif* or titer* or titre*)) or (predict* adj3 (immun* or protect*)) or "protective immunity" or "surrogate endpoint*").ti,ab,hw,tw,mc. or (protection or protective or protected or ((antibody or antibodies) adj2 (status or level*))).ti.	24995
7	5 and 6	15896
8	(1 or 4) and 7	578
9	2 and 7	1229
10	8 or 9	1230
11	limit 10 to yr="2019 -Current"	1191
12	limit 11 to (english or french)	1178
13	(((previous* or prior or past or post or postcovid* or post-covid* or postsars* or post-sars* or after or following or recover* or convalescen* or "after infection") adj3 infect*) or re-infect* or reinfect* or "after first infection").ti,ab,hw,tw,mc.	9662
14	((vaccin* or immunis* or immuniz*) adj3 fail*).ti,ab,hw,tw,mc.	227
15	(((breakthrough* or "break through*") adj5 (vaccin* or infection*)) or "breakthrough rate*").ti,ab,hw,tw,mc.	125
16	(("second dose" or "two doses" or "fully vaccinated") adj5 (infected or infection* or contracted or acquired or acquisition or caught or got* or transmi* or spread* or outbreak* or cluster* or communicab* or contagious* or epidemic* or occurrence or case or cases)).ti,ab,hw,tw,mc.	40
17	((postvaccin* or postimmuniz* or postimmunis* or "post-vaccin*" or "post-immuniz*" or "post-immunis*") adj5 (infected or infection* or contracted or acquired or acquisition or caught or got* or transmi* or spread* or outbreak* or cluster* or communicab* or contagious* or epidemic* or occurrence or case or cases)).ti,ab,hw,tw,mc.	59
18	(((receiv* or recipient* or complet* or already or post or previously or after or following or "subsequent to" or since or had or despite or "in spite of") adj5 (immunis* or immuniz* or inoculat* or innoculat* or vaccin* or revaccinat* or reimmuniz* or reimmunis* or dose or doses) adj5 (infected or infection* or contracted or acquired or acquisition or caught or got* or transmi* or spread* or outbreak* or cluster* or	1131

	communicab* or contagious* or epidemic* or occurrence or case or cases)) or ((infected or infection* or contracted or acquired or acquisition or caught or got* or transmi* or spread* or outbreak* or cluster* or communicab* or contagious* or epidemic* or occurrence or case or cases) adj5 (receiv* or recipient* or complet* or already or post or previously or after or following or "subsequent to" or since or had or despite or "in spite of") adj5 (immunis* or immuniz* or inoculat* or innoculat* or vaccin* or revaccinat* or reimmuniz* or reimmunis* or dose or doses))).ti,ab,hw,tw,mc.	
19	((vaccinated or immunized or immunised) adj5 (infected or infection* or contracted or acquired or acquisition or caught or got* or transmi* or spread* or outbreak* or cluster* or communicab* or contagious* or epidemic* or occurrence or case or cases)).ti,ab,hw,tw,mc.	512
20	((vaccinated or immunized or immunised) adj10 (person or persons or people or man or men or woman or women or adult or adults or patient or patients or individual or individuals or responder or responders or practitioner* or staff or personnel or worker* or employee* or provider* or technician* or clinician* or doctor* or nurse or nurses or paramedic* or physician* or hospitalist* or pharmacist*) adj10 (infected or infection* or contracted or acquired or acquisition or caught or got* or transmi* or spread* or outbreak* or cluster* or communicab* or contagious* or epidemic* or occurrence or case or cases)).ti,ab,hw,tw,mc.	204
21	((escape or escapes or escaped or escaping or evade or evades or evaded or evading or evasion) adj5 ("Ad26.COV2.S" or AZD1222 or BNT162* or ChAdOx1* or Comirnaty or immunis* or immuniz* or "JNJ-78436735" or "mrna-1273*" or Novavax or NVX-CoV2373 or PittCoVacc* or postvaccin* or postimmunis* or postimmuniz* or reimmunis* or reimmuniz* or revacciat* or tozinameran or vaccin*)).ti,ab,hw,tw,mc.	132
22	14 or 15 or 16 or 17 or 18 or 19 or 20 or 21	2046
23	(wane or wanes or waned or waning).ti,hw,tw.	445
24	((decay* or declin* or decreas* or impair* or duration or fail* or fall* or "go* down" or lack or lacks or lacked or lacking or lose or loses or losing or loss or lost or low or lower* or lesser* or poor or poorer* or "adversely affect*" or reduc*) adj5 (protect* or effectiv* or immunity or "immune respon*" or antibod* or sero*)).ti,hw,tw. and (infected or infection* or contracted or acquired or acquisition or caught or got* or transmi* or spread* or outbreak* or cluster* or communicab* or contagious* or epidemic* or occurrence or case or cases).ti,ab,hw,tw,mc.	9420
25	((decay* or declin* or decreas* or impair* or duration or fail* or fall* or "go* down" or lack or lacks or lacked or lacking or lose or loses or losing or loss or lost or low or lower* or lesser* or poor or poorer* or "adversely affect*" or reduc*) adj5 (protect* or effectiv* or immunity or "immune respon*" or antibod* or sero*)).ti,ab,hw,tw,mc. and (infected or infection* or contracted or acquired or acquisition or caught or got* or	9420

	transmi* or spread* or outbreak* or cluster* or communicab* or contagious* or epidemic* or occurrence or case or cases).hw,ti,tw.	
26	23 or 24 or 25	9779
27	(1 or 4) and (13 or 22 or 26) and (5 and 6)	202
28	2 and (13 or 26) and (5 and 6)	366
29	27 or 28	379
30	limit 29 to yr="2019 -Current"	363
31	limit 30 to (english or french)	359
32	12 not 31	819

SCOPUS

#	Queries	Results
#1	TITLE-ABS ("Ad5-nCoV vaccine" OR "BNT162 vaccine" OR "ChAdOx1 COVID-19 vaccine" OR "Covid-19 aAPC vaccine" OR "lentiviral minigene vaccine of COVID-19 cORonavirus" OR "mRNA-1273 vaccine" OR "recombinant SARS-CoV-2 vaccine NVX-cov2373" OR "SARS-CoV-2 inactivated vaccines") OR TITLE-ABS (bnt162* OR comirnaty OR coronavac OR covishield OR tozinameran OR "mrna-1273*" OR chadox1* OR azd1222 OR "Ad26.COV2.S" OR "JNJ-78436735" OR novavax OR nvx-cov2373 OR ad5-ncov OR aapc OR "lentiviral minigene" OR vaxzevria)	1,643
#2	(TITLE-ABS ("Severe acute respiratory syndrome coronavirus 2" OR "coronavirus disease 2019" OR "COVID-19" OR "SARS-CoV-2")) OR (TITLE-ABS ("2019 corona virus" OR "2019 coronavirus" OR "2019 ncov" OR "corona virus 19" OR "corona virus 2019" OR "corona virus disease 19" OR "corona virus disease 2019" OR "corona virus epidemic*" OR "corona virus outbreak*" OR "corona virus pandemic*" OR "corona virus response" OR "coronavirus 19" OR "coronavirus 2019" OR "coronavirus disease 19" OR "coronavirus disease 2019" OR "coronavirus epidemic*" OR "coronavirus outbreak*" OR "coronavirus pandemic*" OR "coronavirus response" OR "covid 19" OR "covid 2019" OR "new corona virus" OR "new coronavirus" OR "novel corona virus" OR "novel coronavirus" OR "novel human coronavirus" OR "sars coronavirus 2" OR "sars cov 2" OR "sars cov2" OR "sars like coronavirus" OR "severe acute respiratory syndrome corona virus 2" OR "severe acute respiratory syndrome coronavirus 2" OR "severe specific contagious pneumonia" OR "wuhan corona virus" OR "wuhan coronavirus" OR ((pandemic* OR novel OR wuhan) W/3 (coronavirus* OR "corona virus*" OR betacoronavirus* OR "beta coronavirus*" OR "beta corona virus*" OR pneumonia* OR sars OR "severe acute respiratory syndrome")) OR (pneumonia W/3 (coronavirus* OR "corona virus*" OR betacoronavirus* OR "beta coronavirus*" OR "beta corona virus*" OR sars OR "severe acute respiratory syndrome")) OR 2019ncov OR covid OR covid19 OR covid2019 OR ncov OR sarscov2)) OR (TITLE ("SARS-Cov-2 variant*" OR vui-202012-01 OR "VOC-202012/01" OR "B.1.1.7" OR b117 OR "B.1.351" OR b1351 OR "B.1.617" OR b1617 OR "P.1" OR p1 OR coronavirus* OR "corona virus*" OR betacoronavirus* OR "beta coronavirus*" OR "beta corona virus*" OR pneumonia* OR sars OR "severe acute respiratory syndrome") OR TITLE ((covid* OR sars* OR variant*) AND (alpha OR beta OR gamma OR delta OR "variant* of concern" OR "VOC")))	346,981

#3	TITLE-ABS ((vaccin* OR immunis* OR immuniz* OR innoculat* OR inoculat* OR jab* OR shot* OR injection* OR nonimmunis* OR nonimmuniz* OR postvaccin* OR postimmunis* OR postimmuniz* OR reimmunis* OR reimmuniz* OR revaccinat* OR unimmunis* OR unimmuniz* OR ((one OR single OR two OR double OR first OR second OR partial* OR full) W/2 (dose* OR injection* OR injected OR injecting)) OR single-dose* OR double-dose* OR bnt162* OR comirnaty OR coronavac OR covishield OR tozinameran OR "mrna-1273*" OR chadox1* OR azd1222 OR "Ad26.COV2.S" OR "JNJ-78436735" OR novavax OR nvx-cov2373 OR ad5-ncov OR aapc OR "lentiviral minigene" OR vaxzevria))	2,005,732
#4	#2 AND #3	25,288
#5	TITLE-ABS (antibody OR antibodies OR antibody-positiv* OR antibody-negativ* OR "complement activation" OR ((humoral OR humoural OR adaptive OR antibody-mediated) W/3 (respon* OR immun*)) OR immunity OR "immune system*" OR "immune marker*" OR ((immun* OR sero*) W/3 respon*) OR immunolog* OR immunomodulat* OR b-lymphocyte* OR "B-cell*") OR TITLE (immun* OR immunoglobulin* OR iga OR igd OR ige OR igg OR igm OR sero* OR sera*)	2,431,187
#6	TITLE-ABS ("correlat* of protection" OR "correlat* with protection" OR "immune correlat*" OR "correlat* of immunity" OR "correlat* of neutraliz*" OR "correlat* of neutralis*" OR "measur* of protection" OR "correlat* of infectivity" OR "protective correlat*" OR ((protective OR protection) W/5 (level* OR threshold* OR marker* OR limit* OR amount* OR mean* OR baseline OR quantif*)) OR ((correlat* OR marker* OR threshold* OR indicat* OR measur*) W/5 (immun* OR protect*)) OR ((antibody OR antibodies OR titer* OR titre*) W/3 (correlat* OR threshold*)) OR (protect* W/5 (immunity OR antibod* OR titer* OR titre*)) OR ((neutraliz* OR neutralis*) W/2 (level* OR amount* OR limit* OR mean* OR baseline OR quantif* OR titer* OR titre*)) OR (predict* W/3 (immun* OR protect*)) OR "protective immunity" OR "surrogate endpoint*") OR TITLE (protection OR protective OR protected OR ((antibody OR antibodies) W/2 (status OR level*)))	678,074
#7	#5 AND #6	200,136
#8	(#1 OR #4) AND #7	1,855
#9	#2 AND #7	3,822
#10	#8 OR #9	3,847
#11	LANGUAGE(English) OR LANGUAGE(French) AND PUBYEAR > 2018	8,905,122
#12	#10 AND #11	2,219
#13	#12 AND NOT INDEX (MEDLINE)	692

LITERATURE SEARCH

09/05/2021

COVID-19 antibody correlates of protection

Preprints

Request prepared by: Library Services
Contact information: library@oahpp.ca

Search results reporting

DATABASES SEARCHED

Database	Date searched	Records	Duplicates removed by database	Remaining
NIH iSearch COVID-19 Portfolio	09/05/2021		n/a	512

RECORDS TOTALS

Records source	Records
Records identified through database searching	512
Duplicates removed (manual de-duping)	38
Total records after duplicates removed	474

Search strategies

National Institutes of Health iSearch COVID-19 Portfolio (Preprints)
https://icite.od.nih.gov/covid19/search/

#	Searches	Results
1	(title:antibody OR title:antibodies OR title:antibody-response OR title:humoral OR title:humoural OR title:titer* OR title:titre* OR title:immune OR title:immunity OR title:"sero* response"~3 OR title:immunolog* OR title:immunomodulat* OR title:immunoglobulin* OR title:B-cell OR title:B-lymphocyte OR title:neutraliz* OR title:neutralis*) AND (title:protect* OR title:threshold* OR title:correlat*) AND PubTypes:"Preprint"	188
2	(title:immun* correlat* OR title:correlat* neutraliz* OR title:correlat* neutralis* OR title:"measur* of protection" OR title:"correlat* of infectivity" OR title:correlat*	220

	protect* OR title:protect* correlat* OR title:protect* threshold* OR title:protect* immunity OR title:predict* immun*) AND (title:antibody OR title:antibodies OR title:antibody-respons* OR title:humoral OR title:humoural OR title:titer* OR title:neutraliz* OR title:neutralis*) AND PubTypes:"Preprint"	
3	(title:reinfect* OR title:re-infect* OR title:"after first infection" OR title:"postcovid* infect*"~5 OR title:"post-covid infect*"~5 OR title:postsars* OR title:"reocurr* infect*"~2) AND (title:protect* OR title:immun* OR title:sero* OR title:sera* OR title:antibod* OR title:titer* OR title:titre* OR title:neutraliz* OR title:neutralis* OR title:humoral OR title:humoural OR title:lymphocyte* OR title:B-cell* OR title:correlate* OR title:threshold* OR title:infectivity) AND PubTypes:"Preprint"	23
4	(title:previous* OR title:prior OR title:past OR title:post OR title:after OR title:following OR title:recover* OR title:convalescen*) AND (title:infect*) AND (title:reinfect* OR title:re-infect*) AND PubTypes:"Preprint"	7
5	(title:postcovid OR title:post-covid OR title:postsars OR title:post-sars) AND (title:infect* OR title:reinfect* OR title:re-infect*) AND PubTypes:"Preprint"	6
6	(title:breakthrough* infection* OR title:"break through* infection*"~5 OR title:breakthrough* rate* OR title:breakthrough* vaccin* OR title:"break through* vaccin*"~5) AND (title:protect* OR title:immun* OR title:sero* OR title:sera* OR title:antibod* OR title:titer* OR title:titre* OR title:neutraliz* OR title:neutralis* OR title:humoral OR title:humoural OR title:lymphocyte* OR title:B-cell* OR title:correlate* OR title:threshold* OR title:infectivity) AND PubTypes:"Preprint"	9
7	(title:"one dose" OR title:"single vaccin*" OR title:"two dose*" OR title:"double dose*" OR title:"double vaccin*" OR title:"second dose" OR title:"two doses" OR title:"fully vaccinated" OR title:"partial* vaccin*" OR title:vaccinated OR title:immunized OR title:immunised OR title:postvaccin* OR title:postimmuniz* OR title:postimmunis* OR title:post-vaccin* OR title:post-immuniz* OR title:post-immunis*) AND (title:infect* OR title:re-infect* OR title:reinfect* OR title:"after first infection" OR title:contract* OR title:transmi* OR title:spread* OR title:outbreak* OR title:case* OR title:cluster* OR title:communicab* OR title:contagious*) AND PubTypes:"Preprint"	35
8	(title:escap* OR title:evad* OR title:evasion) AND (title:"Ad26.COV2.S" OR title:AZD1222 OR title:BNT162* OR title:ChAdOx1* OR title:Comirnaty OR title:immunis* OR title:immuniz* OR title:"JNJ-78436735" OR title:"mrna-1273*" OR title:Novavax OR title:NVX-CoV2373 OR title:PittCoVacc* OR title:postvaccin* OR title:postimmunis* OR title:postimmuniz* OR title:reimmunis* OR title:reimmuniz* OR title:revacciat* OR title:tozinameran OR title:vaccin*) AND PubTypes:"Preprint"	24

Search results

Results attached as a CSV Excel file

LITERATURE SEARCH

10/05/2021

COVID-19 antibody correlates of protection

Preprints (supplemental search)

Request prepared by: Library Services
Contact information: library@oahpp.ca

Search results reporting

DATABASES SEARCHED

Database	Date searched	Records	Duplicates removed by database	Remaining
NIH iSearch COVID-19 Portfolio	10/05/2021		n/a	194

RECORDS TOTALS

Records source	Records
Records identified through database searching	194
Duplicates removed (manual de-duping)	55
Total records after duplicates removed	139

Search strategies

National Institutes of Health iSearch COVID-19 Portfolio (Preprints)
https://icite.od.nih.gov/covid19/search/

#	Searches	Results
1	(title:correlat* of protect*) AND PubTypes:"Preprint"	10
2	(abstract:"correlate of protection") OR (abstract:"correlates of protection") AND PubTypes:"Preprint"	51
3	(supplemental text:"correlate of protection") OR (supplemental text:"correlates of protection") AND PubTypes:"Preprint"	20
4	(title:correlate* of immun*) OR (title:correlate* of neutraliz*) AND PubTypes:"Preprint"	18

5	(title:protect* neutraliz*) OR (title:protect* immune*) OR (title:protect* threshold*) OR (title:neutraliz* threshold*) OR (title:antibod* threshold*) OR (title:titer* threshold*) OR (title:protect* level*) OR (title:optimal protection) OR (title:protect* correlate*) OR (title:immune* correlat*) AND PubTypes:"Preprint"	95

Search results

Results attached as a CSV Excel file

COVID-19 antibody correlates of protection

Preprints search update (original search run 2021 Oct 5th)

Request prepared by: Library Services
Contact information: library@oahpp.ca

Search results reporting

DATABASES SEARCHED

Database	Date searched	Records	Duplicates removed by database	Remaining
NIH iSearch COVID-19 Portfolio	12/31/2021	27	n/a	27

RECORDS TOTALS

Records source	Records
Records identified through database searching	27
Duplicates removed (manual de-duping)	7
Total records after duplicates removed	20

Search strategies

National Institutes of Health iSearch COVID-19 Portfolio (Preprints)
https://icite.od.nih.gov/covid19/search/

#	Searches	Results
1	(title:correlat* of protect*) AND PubTypes:"Preprint" AND Publication Date: 2021-10-06-*	2
2	(abstract:"correlate of protection") OR (abstract:"correlates of protection") AND PubTypes:"Preprint" AND Publication Date: 2021-10-06-*	11
3	(supplemental text:"correlate of protection") OR (supplemental text:"correlates of protection") AND PubTypes:"Preprint" AND Publication Date: 2021-10-06-*	2
4	(title:correlate* of immun*) OR (title:correlate* of neutraliz*) AND PubTypes:"Preprint" AND Publication Date: 2021-10-06-*	1

5	(title:protect* neutraliz*) OR (title:protect* immune*) OR (title:protect* threshold*) OR (title:neutraliz* threshold*) OR (title:antibod* threshold*) OR (title:titer* threshold*) OR (title:protect* level*) OR (title:optimal protection) OR (title:protect* correlate*) OR (title:immune* correlat*) AND PubTypes:"Preprint" AND Publication Date: 2021-10-06-*	11

Search results

Results attached as a CSV Excel file

LITERATURE SEARCH

12/31/2021

COVID-19 antibody correlates of protection

Preprints search update (original search run 2021 Sept 3rd)

Request prepared by: Library Services
Contact information: library@oahpp.ca

Search results reporting

DATABASES SEARCHED

Database	Date searched	Records	Duplicates removed by database	Remaining
NIH iSearch COVID-19 Portfolio	12/31/2021	161	n/a	161

RECORDS TOTALS

Records source	Records
Records identified through database searching	161
Duplicates removed (manual de-duping)	11
Total records after duplicates removed	150

Search strategies

National Institutes of Health iSearch COVID-19 Portfolio (Preprints)
https://icite.od.nih.gov/covid19/search/

#	Searches	Results
1	(title:antibody OR title:antibodies OR title:antibody-response OR title:humoral OR title:humoural OR title:titer* OR title:titre* OR title:immune OR title:immunity OR title:"sero* response"~3 OR title:immunolog* OR title:immunomodulat* OR title:immunoglobulin* OR title:B-cell OR title:B-lymphocyte OR title:neutraliz* OR title:neutralis*) AND (title:protect* OR title:threshold* OR title:correlat*) AND PubTypes:"Preprint" AND Publication Date: 2021-09-04-*	28
2	(title:immun* correlat* OR title:correlat* neutraliz* OR title:correlat* neutralis* OR title:"measur* of protection" OR title:"correlat* of infectivity" OR title:correlat* protect* OR title:protect* correlat* OR title:protect* threshold* OR title:protect* immunity OR title:predict* immun*) AND (title:antibody OR title:antibodies OR title:antibody-respons* OR title:humoral OR title:humoural OR title:titer* OR title:neutraliz* OR title:neutralis*) AND PubTypes:"Preprint" AND Publication Date: 2021-09-04-*	68
3	(title:reinfect* OR title:re-infect* OR title:"after first infection" OR title:"postcovid* infect*"~5 OR title:"post-covid infect*"~5 OR title:postsars* OR title:"reocurr* infect*"~2) AND (title:protect* OR title:immun* OR title:sero* OR title:sera* OR title:antibod* OR title:titer* OR title:titre* OR title:neutraliz* OR title:neutralis* OR title:humoral OR title:humoural OR title:lymphocyte* OR title:B-cell* OR title:correlate* OR title:threshold* OR title:infectivity) AND PubTypes:"Preprint" AND Publication Date: 2021-09-04-*	3
4	(title:previous* OR title:prior OR title:past OR title:post OR title:after OR title:following OR title:recover* OR title:convalescen*) AND (title:infect*) AND (title:reinfect* OR title:re-infect*) AND PubTypes:"Preprint" AND Publication Date: 2021-09-04-*	0
5	(title:postcovid OR title:post-covid OR title:postsars OR title:post-sars) AND (title:infect* OR title:reinfect* OR title:re-infect*) AND PubTypes:"Preprint" AND Publication Date: 2021-09-04-*	2
6	(title:breakthrough* infection* OR title:"break through* infection*"~5 OR title:breakthrough* rate* OR title:breakthrough* vaccin* OR title:"break through* vaccin*"~5) AND (title:protect* OR title:immun* OR title:sero* OR title:sera* OR title:antibod* OR title:titer* OR title:titre* OR title:neutraliz* OR title:neutralis* OR title:humoral OR title:humoural OR title:lymphocyte* OR title:B-cell* OR title:correlate* OR title:threshold* OR title:infectivity) AND PubTypes:"Preprint" AND Publication Date: 2021-09-04-*	21
7	(title:"one dose" OR title:"single vaccin*" OR title:"two dose*" OR title:"double dose*" OR title:"double vaccin*" OR title:"second dose" OR title:"two doses" OR title:"fully vaccinated" OR title:"partial* vaccin*" OR title:vaccinated OR title:immunized OR title:immunised OR title:postvaccin* OR title:postimmuniz* OR title:postimmunis* OR title:post-vaccin* OR title:post-immuniz* OR title:post-immunis*) AND (title:infect* OR title:re-infect* OR title:reinfect* OR title:"after first infection" OR title:contract* OR title:transmi* OR title:spread* OR title:outbreak* OR title:case* OR title:cluster* OR title:communicab* OR title:contagious*) AND PubTypes:"Preprint" AND Publication Date: 2021-09-04-*	27
8	(title:escap* OR title:evad* OR title:evasion) AND (title:"Ad26.COV2.S" OR title:AZD1222 OR title:BNT162* OR title:ChAdOx1* OR title:Comirnaty OR title:immunis* OR title:immuniz* OR title:"JNJ-78436735" OR title:"mrna-1273*" OR title:Novavax OR title:NVX-CoV2373 OR title:PittCoVacc* OR title:postvaccin* OR title:postimmunis* OR title:postimmuniz* OR title:reimmunis* OR title:reimmuniz* OR	12

title:revacciat* OR title:tozinameran OR title:vaccin*) AND PubTypes:"Preprint" AND Publication Date: 2021-09-04-*

Search results

Results attached as a CSV Excel file

LITERATURE SEARCH

12/31/2021

COVID-19 antibody correlates of protection (revised) – Search update: 2021 Dec 31

Request prepared by: Library Services
Contact information: library@oahpp.ca

Search results reporting

DATABASES SEARCHED

Database	Date searched	Records	Duplicates removed by database	Remaining
MEDLINE (I)	12/31/2021	960	0	960
MEDLINE (II)	12/31/2021	438	0	438

RESULTS TOTALS

Records source	Records
Records identified through database searching	1398
Duplicates removed by database	0
Duplicates removed by bibliographic management software	409
Total records after duplicates removed	989

Search strategies

MEDLINE

Ovid MEDLINE(R) ALL <1946 to December 30, 2021>

#	Searches	Results
1	COVID-19 Vaccines/ or (ncov or BNT162* or "mrna-1273").rn,nm,os,ox,ps,px,rs,rx. or ("Ad5-nCoV vaccine" or "BNT162 vaccine" or "ChAdOx1 COVID-19 vaccine" or "Covid-19 aAPC vaccine" or "lentiviral minigene vaccine of COVID-19 coronavirus" or "mRNA-1273 vaccine" or "recombinant SARS-CoV-2 vaccine NVX-cov2373" or "SARS-CoV-2 inactivated vaccines").rn. or (BNT162* or Comirnaty or CoronaVac or Covishield or Tozinameran or "mrna-1273*" or ChAdOx1* or AZD1222 or "Ad26.COV2.S" or "JNJ-78436735" or Novavax or NVX-CoV2373 or Ad5-nCoV or aAPC or "lentiviral minigene" or vaxzevria).ti,kf,kw.	8487
2	COVID-19/ or SARS-CoV-2/ or "SARS-CoV-2 variants".os. or "COVID-19 breakthrough infections".rs. or ("COVID-19" or "SARS-CoV-2").nm,os,ox,ps,px,rs,rs. or (Pandemics/ and (Coronavirus Infections/ or Betacoronavirus/ or Spike Glycoprotein, Coronavirus/ or Pneumonia, Viral/)) or exp COVID-19 Testing/ or COVID-19 Serological Testing/ or ("2019 corona virus" or "2019 coronavirus" or "2019 ncov" or "corona virus 19" or "corona virus 2019" or "corona virus disease 19" or "corona virus disease 2019" or "corona virus epidemic*" or "corona virus outbreak*" or "corona virus pandemic*" or "corona virus response" or "coronavirus 19" or "coronavirus 2019" or "coronavirus disease 19" or "coronavirus disease 2019" or "coronavirus epidemic*" or "coronavirus outbreak*" or "coronavirus pandemic*" or "coronavirus response" or "covid 19" or "covid 2019" or "new corona virus" or "new coronavirus" or "novel corona virus" or "novel coronavirus" or "novel human coronavirus" or "sars coronavirus 2" or "sars cov 2" or "sars cov2" or "sars like coronavirus" or "severe acute respiratory syndrome corona virus 2" or "severe acute respiratory syndrome coronavirus 2" or "severe specific contagious pneumonia" or "wuhan corona virus" or "wuhan coronavirus" or ((pandemic* or novel or wuhan or delta) adj3 (coronavirus* or "corona virus*" or betacoronavirus* or "beta coronavirus*" or "beta corona virus*" or pneumonia* or SARS or "severe acute respiratory syndrome")) or (pneumonia adj3 (coronavirus* or "corona virus*" or betacoronavirus* or "beta coronavirus*" or "beta corona virus*" or SARS or "severe acute respiratory syndrome")) or 2019ncov or covid or covid19 or covid2019 or ncov or sarscov2).kf,kw,ti. or (coronavirus* or "corona virus*" or betacoronavirus* or "beta coronavirus*" or "beta corona virus*" or pneumonia* or SARS or "severe acute respiratory syndrome").ti. or ("2019 corona virus" or "2019 coronavirus" or "2019 ncov" or "corona virus 19" or "corona virus 2019" or "corona virus disease 19" or "corona virus disease 2019" or "corona virus epidemic*" or "corona virus outbreak*" or "corona virus pandemic*" or "corona virus response" or "coronavirus 19" or "coronavirus 2019" or "coronavirus disease 19" or "coronavirus	294391

	disease 2019" or "coronavirus epidemic*" or "coronavirus outbreak*" or "coronavirus pandemic*" or "coronavirus response" or "covid 19" or "covid 2019" or "new corona virus" or "new coronavirus" or "novel corona virus" or "novel coronavirus" or "novel human coronavirus" or "sars coronavirus 2" or "sars cov 2" or "sars cov2" or "sars like coronavirus" or "severe acute respiratory syndrome corona virus 2" or "severe acute respiratory syndrome coronavirus 2" or "severe specific contagious pneumonia" or "wuhan corona virus" or "wuhan coronavirus" or ((pandemic* or novel or wuhan or delta) adj3 (coronavirus* or "corona virus*" or betacoronavirus* or "beta coronavirus*" or "beta corona virus*" or pneumonia* or SARS or "severe acute respiratory syndrome")) or (pneumonia adj3 (coronavirus* or "corona virus*" or betacoronavirus* or "beta coronavirus*" or "beta corona virus*" or SARS or "severe acute respiratory syndrome")) or 2019ncov or covid or covid19 or covid2019 or ncov or sarscov2).ab. /freq=2	
3	(Omicron or "B.1.1.529" or "VOC-21NOV-01" or "B.1.1.529.1" or "B.1.1.529.2" or B11529*).ab,ti,kw,kf.	501
4	2 or 3	294776
5	Immunization Programs/ or Immunization Schedule/ or Immunization, Secondary/ or Immunization, Passive/ or Immunization/ or Immunogenicity, Vaccine/ or Immunotherapy, Active/ or Mass Vaccination/ or Vaccination Coverage/ or Vaccination/ or Vaccines, Attenuated/ or Vaccines, Combined/ or Vaccines, Synthetic/ or Vaccines/ or Viral Vaccines/ or COVID-19 Vaccines/ or vaccine*.rn. or (vaccin* or immunis* or immuniz* or innoculat* or inoculat* or jab* or shot* or injection* or nonimmunis* or nonimmuniz* or postvaccin* or postimmunis* or postimmuniz* or reimmunis* or reimmuniz* or revaccinat* or unimmunis* or unimmuniz* or ((one or single two or double or first or second or partial* or full) adj2 (dose* or injection* or injected or injecting)) or single-dose* or double-dose* or BNT162* or Comirnaty or CoronaVac or Covishield or Tozinameran or "mrna-1273*" or ChAdOx1* or AZD1222 or "Ad26.COV2.S" or "JNJ-78436735" or Novavax or NVX-CoV2373 or Ad5-nCoV or aAPC or "lentiviral minigene" or vaxzevria).ti,ab,kf,kw.	1302150
6	4 and 5	33920
7	Adaptive Immunity/ or Antibodies, Neutralizing/ or Antibodies, Viral/ or Antibody Affinity/ or Antibody Formation/ or Antibody Specificity/ or Antibodies, Monoclonal/ or Binding Sites, Antibody/ or exp Antibodies/ or exp Antigen-Antibody Reactions/ or Antibody-Dependent Enhancement/ or Antibody-Dependent Cell Cytotoxicity/ or Lymphocytes/ or exp B-Lymphocytes/ or B-Lymphocyte subsets/ or Lymphocyte Activation/ or Complement Activation/ or Th2 Cells/ or Immune System Phenomena/ or Immunity/ or Immunity, Active/ or Immunity, Humoral/ or Immunity, Mucosal/ or Immunologic Factors/ or Immunologic Surveillance/ or Immunity, Innate/ or Immunity, Heterologous/ or Immunity, Maternally-Acquired/ or Immune Sera/ or exp Immunoglobulins/ or Immunoglobulin M/ or Immunoglobulin G/ or exp	2833106

	Immunomodulation/ or Immunologic Memory/ or Models, Immunological/ or Cross Protection/im or Cross Reactions/im or Reinfection/im or Recurrence/im or "Severity of Illness Index"/im or Viral Load/im or Convalescence/im or Seroconversion/ or Seroepidemiologic Studies/ or Neutralization Tests/ or Enzyme-Linked Immunosorbent Assay/ or Protein Domains/im or Receptors, Virus/im or Spike Glycoprotein, Coronavirus/im or Coronavirus Nucleocapsid Proteins/im or *COVID-19 Vaccines/im or *COVID-19/im or *SARS-CoV-2/im or (antibody or antibodies or antibody-positiv* or antibody-negativ* or "complement activation" or ((humoral or humoural or adaptive or antibody-mediated) adj3 (respon* or immun*)) or immunity or "immune system*" or "immune marker*" or ((immun* or sero*) adj3 respon*) or immunolog* or immunomodulat* or B-lymphocyte* or "B-cell*").ti,ab,kf,kw. or (immun* or immunoglobulin* or IgA or IgD or IgE or IgG or IgM or sero* or sera*).ti,kf,kw.	
8	("correlat* of protection" or "correlat* with protection" or "immune correlat*" or "correlat* of immunity" or "correlat* of neutraliz*" or "correlat* of neutralis*" or "measur* of protection" or "correlat* of infectivity" or "protective correlat*" or ((protective or protection) adj5 (level* or threshold* or marker* or limit* or amount* or mean* or baseline or quantif*)) or ((correlat* or marker* or threshold* or indicat* or measur*) adj5 (immun* or protect*)) or ((antibody or antibodies or titer* or titre*) adj3 (correlat* or threshold*)) or (protect* adj5 (immunity or antibod* or titer* or titre*)) or ((neutraliz* or neutralis*) adj2 (level* or amount* or limit* or mean* or baseline or quantif* or titer* or titre*)) or (predict* adj3 (immun* or protect*)) or "protective immunity" or "surrogate endpoint*").ti,ab,kf,kw. or (protection or protective or protected or ((antibody or antibodies) adj2 (status or level*))).ti.	378764
9	(1 or 6) and 7 and 8	2767
10	4 and 7 and 8	4896
11	9 or 10	4902
12	limit 11 to yr="2021 -Current"	2686
13	(202108* or 202109* or 202110* or 202111* or 202112* or 202201*).ez. or (202108* or 202109* or 202110* or 202111* or 202112* or 202201*).dt. or (202108* or 202109* or 202110* or 202111* or 202112* or 202201* or 2021 08* or 2021 09* or 2021 10* or 2021 11* or 2021 12* or 2022 01* or 2021 aug or 2021 sep or 2021 oct or 2021 nov or 2021 dec or 2022 jan).dp.	802962
14	12 and 13	1419
15	limit 14 to (english or french)	1398
16	Reinfection/ or Recurrence/ or Follow-Up Studies/ or ((Convalescence/ or "Severity of Illness Index"/ or Viral Load/ or Asymptomatic Infections/) and (re-infect* or reinfect*).ti,kf,kw.)	838483
17	(((previous* or prior or past or post or postcovid* or post-covid* or postsars* or post-sars* or after or following or recover*) adj2 infect*) or re-infect* or reinfect* or "after	139085

	first infection" or "after infection").ti,ab,kf,kw. or (convalescence or convalescent).ti,kf,kw.	
18	16 or 17	969761
19	Treatment Failure/ or ((vaccin* or immunis* or immuniz*) adj3 fail*).ab,kf,kw,ti.	39893
20	(((breakthrough* or "break through*") adj5 (vaccin* or infection*)) or "breakthrough rate*").ab,kf,kw,ti.	1599
21	(("second dose" or "two doses" or "fully vaccinated") adj5 (infected or infection* or contracted or acquired or acquisition or caught or got* or transmi* or spread* or outbreak* or cluster* or communicab* or contagious* or epidemic* or occurrence or case or cases)).ab,kf,kw,ti.	481
22	((postvaccin* or postimmuniz* or postimmunis* or "post-vaccin*" or "post-immuniz*" or "post-immunis*") adj5 (infected or infection* or contracted or acquired or acquisition or caught or got* or transmi* or spread* or outbreak* or cluster* or communicab* or contagious* or epidemic* or occurrence or case or cases)).ab,kf,kw,ti.	677
23	(((receiv* or recipient* or complet* or already or post or previously or after or following or "subsequent to" or since or had or despite or "in spite of") adj5 (immunis* or immuniz* or inoculat* or innoculat* or vaccin* or revaccinat* or reimmuniz* or reimmunis* or dose or doses) adj5 (infected or infection* or contracted or acquired or acquisition or caught or got* or transmi* or spread* or outbreak* or cluster* or communicab* or contagious* or epidemic* or occurrence or case or cases)) or ((infected or infection* or contracted or acquired or acquisition or caught or got* or transmi* or spread* or outbreak* or cluster* or communicab* or contagious* or epidemic* or occurrence or case or cases) adj5 (receiv* or recipient* or complet* or already or post or previously or after or following or "subsequent to" or since or had or despite or "in spite of") adj5 (immunis* or immuniz* or inoculat* or innoculat* or vaccin* or revaccinat* or reimmuniz* or reimmunis* or dose or doses))).ab,kf,kw,ti.	19960
24	((vaccinated or immunized or immunised) adj5 (infected or infection* or contracted or acquired or acquisition or caught or got* or transmi* or spread* or outbreak* or cluster* or communicab* or contagious* or epidemic* or occurrence or case or cases)).ab,kf,kw,ti.	8870
25	((vaccinated or immunized or immunised) adj10 (person or persons or people or man or men or woman or women or adult or adults or patient or patients or individual or individuals or responder or responders or practitioner* or staff or personnel or worker* or employee* or provider* or technician* or clinician* or doctor* or nurse or nurses or paramedic* or physician* or hospitalist* or pharmacist*) adj10 (infected or infection* or contracted or acquired or acquisition or caught or got* or transmi* or spread* or outbreak* or cluster* or communicab* or contagious* or epidemic* or occurrence or case or cases)).ab,kf,kw,ti.	3016

26	((escape or escapes or escaped or escaping or evade or evades or evaded or evading or evasion) adj5 ("Ad26.COV2.S" or AZD1222 or BNT162* or ChAdOx1* or Comirnaty or immunis* or immuniz* or "JNJ-78436735" or "mrna-1273*" or Novavax or NVX-CoV2373 or PittCoVacc* or postvaccin* or postimmunis* or postimmuniz* or reimmunis* or reimmuniz* or revacciat* or tozinameran or vaccin*)).ab,kf,kw,ti.	1081
27	19 or 20 or 21 or 22 or 23 or 24 or 25 or 26	69543
28	(wane or wanes or waned or waning).kf,kw,ti.	606
29	((decay* or declin* or decreas* or impair* or duration or fail* or fall* or "go* down" or lack or lacks or lacked or lacking or lose or loses or losing or loss or lost or low or lower* or lesser* or poor or poorer* or "adversely affect*" or reduc*) adj5 (protect* or effectiv* or immunity or "immune respon*" or antibod* or sero*)).kf,kw,ti. and (infected or infection* or contracted or acquired or acquisition or caught or got* or transmi* or spread* or outbreak* or cluster* or communicab* or contagious* or epidemic* or occurrence or case or cases).ab,kf,kw,ti.	6314
30	((decay* or declin* or decreas* or impair* or duration or fail* or fall* or "go* down" or lack or lacks or lacked or lacking or lose or loses or losing or loss or lost or low or lower* or lesser* or poor or poorer* or "adversely affect*" or reduc*) adj5 (protect* or effectiv* or immunity or "immune respon*" or antibod* or sero*)).ab,kf,kw,ti. and (infected or infection* or contracted or acquired or acquisition or caught or got* or transmi* or spread* or outbreak* or cluster* or communicab* or contagious* or epidemic* or occurrence or case or cases).kf,kw,ti.	30642
31	28 or 29 or 30	35386
32	(1 or 6) and (18 or 27 or 31) and (7 and 8)	852
33	4 and (18 or 31) and (7 and 8)	1144
34	32 or 33	1319
35	limit 34 to yr="2021 -Current"	840
36	(202108* or 202109* or 202110* or 202111* or 202112* or 202201*).ez. or (202108* or 202109* or 202110* or 202111* or 202112* or 202201*).dt. or (202108* or 202109* or 202110* or 202111* or 202112* or 202201* or 2021 08* or 2021 09* or 2021 10* or 2021 11* or 2021 12* or 2022 01* or 2021 aug or 2021 sep or 2021 oct or 2021 nov or 2021 dec or 2022 jan).dp.	802962
37	35 and 36	444
38	limit 37 to (english or french)	438
39	15 not 38	960

01/04/2022

COVID-19 antibody correlates of protection (revised): Supplementary Databases

Search update - 2022 Jan 04 (Part 1)

Request prepared by: Library Services
Contact information: library@oahpp.ca

Search results reporting

DATABASES SEARCHED

Database	Date searched	Records	Duplicates removed by database	Remaining
EMBASE	01/04/2022	231	0	231
GLOBAL HEALTH	01/04/2022	424	0	424

RESULTS TOTALS

Records source	Records
Records identified through database searching	655
Duplicates removed by database	0
Duplicates removed by bibliographic management software	521
Total records after duplicates removed	134

Search strategies

EMBASE

Embase <1974 to 2021 Week 52>

#	Searches	Results

1	SARS-CoV-2 vaccine/ or (ncov or BNT162* or "mrna-1273").rn,os,ox,px,rs. or ("Ad5-nCoV vaccine" or "BNT162 vaccine" or "ChAdOx1 COVID-19 vaccine" or "Covid-19 aAPC vaccine" or "lentiviral minigene vaccine of COVID-19 coronavirus" or "mRNA-1273 vaccine" or "recombinant SARS-CoV-2 vaccine NVX-cov2373" or "SARS-CoV-2 inactivated vaccines").rn. or (BNT162* or Comirnaty or CoronaVac or Covishield or Tozinameran or "mrna-1273*" or ChAdOx1* or AZD1222 or "Ad26.COV2.S" or "JNJ-78436735" or Novavax or NVX-CoV2373 or Ad5-nCoV or aAPC or "lentiviral minigene" or vaxzevria).ti,kf,kw.	9147
2	coronavirus disease 2019/ or Severe acute respiratory syndrome coronavirus 2/ or "SARS-CoV-2 variants".os. or "COVID-19 breakthrough infections".rs. or ("COVID-19" or "SARS-CoV-2").os,ox,px,rs,rs. or (pandemic/ and (Coronavirus infection/ or Betacoronavirus/ or coronavirus spike glycoprotein/ or virus pneumonia/)) or exp COVID-19 testing/ or COVID-19 serological testing/ or ("2019 corona virus" or "2019 coronavirus" or "2019 ncov" or "corona virus 19" or "corona virus 2019" or "corona virus disease 19" or "corona virus disease 2019" or "corona virus epidemic*" or "corona virus outbreak*" or "corona virus pandemic*" or "corona virus response" or "coronavirus 19" or "coronavirus 2019" or "coronavirus disease 19" or "coronavirus disease 2019" or "coronavirus epidemic*" or "coronavirus outbreak*" or "coronavirus pandemic*" or "coronavirus response" or "covid 19" or "covid 2019" or "new corona virus" or "new coronavirus" or "novel corona virus" or "novel coronavirus" or "novel human coronavirus" or "sars coronavirus 2" or "sars cov 2" or "sars cov2" or "sars like coronavirus" or "severe acute respiratory syndrome corona virus 2" or "severe acute respiratory syndrome coronavirus 2" or "severe specific contagious pneumonia" or "wuhan corona virus" or "wuhan coronavirus" or ((pandemic* or novel or wuhan or delta) adj3 (coronavirus* or "corona virus*" or betacoronavirus* or "beta coronavirus*" or "beta corona virus*" or pneumonia* or SARS or "severe acute respiratory syndrome")) or (pneumonia adj3 (coronavirus* or "corona virus*" or betacoronavirus* or "beta coronavirus*" or "beta corona virus*" or SARS or "severe acute respiratory syndrome")) or 2019ncov or covid or covid19 or covid2019 or ncov or sarscov2).kf,kw,ti. or (coronavirus* or "corona virus*" or betacoronavirus* or "beta coronavirus*" or "beta corona virus*" or pneumonia* or SARS or "severe acute respiratory syndrome").ti. or ("2019 corona virus" or "2019 coronavirus" or "2019 ncov" or "corona virus 19" or "corona virus 2019" or "corona virus disease 19" or "corona virus disease 2019" or "corona virus epidemic*" or "corona virus outbreak*" or "corona virus pandemic*" or "corona virus response" or "coronavirus 19" or "coronavirus 2019" or "coronavirus disease 19" or "coronavirus disease 2019" or "coronavirus epidemic*" or "coronavirus outbreak*" or "coronavirus pandemic*" or "coronavirus response" or "covid 19" or "covid 2019" or "new corona virus" or "new coronavirus" or "novel corona virus" or "novel coronavirus" or "novel human coronavirus" or "sars coronavirus 2" or "sars cov 2" or "sars cov2" or "sars like	319718

	coronavirus" or "severe acute respiratory syndrome corona virus 2" or "severe acute respiratory syndrome coronavirus 2" or "severe specific contagious pneumonia" or "wuhan corona virus" or "wuhan coronavirus" or ((pandemic* or novel or wuhan or delta) adj3 (coronavirus* or "corona virus*" or betacoronavirus* or "beta coronavirus*" or "beta corona virus*" or pneumonia* or SARS or "severe acute respiratory syndrome")) or (pneumonia adj3 (coronavirus* or "corona virus*" or betacoronavirus* or "beta coronavirus*" or "beta corona virus*" or SARS or "severe acute respiratory syndrome")) or 2019ncov or covid or covid19 or covid2019 or ncov or sarscov2).ab. /freq=2	
3	(Omicron or "B.1.1.529" or "VOC-21NOV-01" or "B.1.1.529.1" or "B.1.1.529.2" or B11529*).ab,ti,kw,kf.	183
4	2 or 3	319871
5	immunization/ or secondary immunization/ or vaccine immunogenicity/ or active immunotherapy/ or mass immunization/ or vaccination/ or vaccination coverage/ or vaccination refusal/ or live vaccine/ or vaccine/ or recombinant vaccine/ or virus vaccine/ or SARS-CoV-2 vaccine/ or (vaccin* or immunis* or immuniz* or innoculat* or inoculat* or jab* or shot* or injection* or nonimmunis* or nonimmuniz* or postvaccin* or postimmunis* or postimmuniz* or reimmunis* or reimmuniz* or revaccinat* or unimmunis* or unimmuniz* or ((one or single two or double or first or second or partial* or full) adj2 (dose* or injection* or injected or injecting)) or single-dose* or double-dose* or BNT162* or Comirnaty or CoronaVac or Covishield or Tozinameran or "mrna-1273*" or ChAdOx1* or AZD1222 or "Ad26.COV2.S" or "JNJ-78436735" or Novavax or NVX-CoV2373 or Ad5-nCoV or aAPC or "lentiviral minigene" or vaxzevria).ti,ab,kw.	1597974
6	4 and 5	36945
7	adaptive immunity/ or neutralizing antibody/ or virus antibody/ or antibody affinity/ or antibody production/ or antibody specificity/ or monoclonal antibody/ or antibody combining site/ or exp antibody/ or exp antigen antibody reaction/ or antibody dependent enhancement/ or antibody dependent cellular cytotoxicity/ or antibody response/ or lymphocyte/ or exp B lymphocyte/ or B lymphocyte subpopulation/ or lymphocyte activation/ or complement activation/ or Th2 cell/ or immunity/ or active immunization/ or humoral immunity/ or mucosal immunity/ or immunologic factor/ or immunosurveillance/ or innate immunity/ or heterologous immunity/ or passive immunization/ or antiserum/ or exp immunoglobulin/ or immunoglobulin G/ or immunological memory/ or biological model/ or cross protection/ or cross reaction/ or reinfection/ or recurrent disease/ or "severity of illness index"/ or virus load/ or convalescence/ or seroconversion/ or seroepidemiology/ or serodiagnosis/ or enzyme linked immunosorbent assay/ or protein domain/ or virus receptor/ or coronavirus spike glycoprotein/ or nucleocapsid protein/ or (antibody or antibodies or antibody-positiv* or antibody-negativ* or "complement activation" or ((humoral or humoural	3993818

	or adaptive or antibody-mediated) adj3 (respon* or immun*)) or immunity or "immune system*" or "immune marker*" or ((immun* or sero*) adj3 respon*) or immunolog* or immunomodulat* or B-lymphocyte* or "B-cell*").ti,kw. or (antibody or antibodies or antibody-positiv* or antibody-negativ* or "complement activation" or ((humoral or humoural or adaptive or antibody-mediated) adj3 (respon* or immun*)) or immunity or "immune system*" or "immune marker*" or ((immun* or sero*) adj3 respon*) or immunolog* or immunomodulat* or B-lymphocyte* or "B-cell*").ab. /freq=2 or (immun* or immunoglobulin* or IgA or IgD or IgE or IgG or IgM or sero* or sera*).ti,kw.	
8	("correlat* of protection" or "correlat* with protection" or "immune correlat*" or "correlat* of immunity" or "correlat* of neutraliz*" or "correlat* of neutralis*" or "measur* of protection" or "correlat* of infectivity" or "protective correlat*" or ((protective or protection) adj5 (level* or threshold* or marker* or limit* or amount* or mean* or baseline or quantif*)) or ((correlat* or marker* or threshold* or indicat* or measur*) adj5 (immun* or protect*)) or ((antibody or antibodies or titer* or titre*) adj3 (correlat* or threshold*)) or (protect* adj5 (immunity or antibod* or titer* or titre*)) or ((neutraliz* or neutralis*) adj2 (level* or amount* or limit* or mean* or baseline or quantif* or titer* or titre*)) or (predict* adj3 (immun* or protect*)) or "protective immunity" or "surrogate endpoint*").ti,ab,kw. or (protection or protective or protected or ((antibody or antibodies) adj2 (status or level*))).ti.	484212
9	7 and 8	228405
10	reinfection/ or recurrent disease/ or ((follow up/ or convalescence/ or "severity of illness index"/ or virus load/ or asymptomatic infection/) and (re-infect* or reinfect*).ti,kw.)	204048
11	(((previous* or prior or past or post or postcovid* or post-covid* or postsars* or post-sars* or after or following or recover* or convalescen* or "after infection") adj2 infect*) or re-infect* or reinfect* or "after first infection").ti,ab,kw.	171733
12	10 or 11	366022
13	treatment failure/ or ((vaccin* or immunis* or immuniz*) adj3 fail*).ab,kw,ti.	146623
14	(((breakthrough* or "break through*") adj5 (vaccin* or infection*)) or "breakthrough rate*").ab,kw,ti.	2130
15	(("second dose" or "two doses" or "fully vaccinated") adj5 (infected or infection* or contracted or acquired or acquisition or caught or got* or transmi* or spread* or outbreak* or cluster* or communicab* or contagious* or epidemic* or occurrence or case or cases)).ab,kw,ti.	581
16	((postvaccin* or postimmuniz* or postimmunis* or "post-vaccin*" or "post-immuniz*" or "post-immunis*") adj5 (infected or infection* or contracted or acquired or acquisition or caught or got* or transmi* or spread* or outbreak* or cluster* or communicab* or contagious* or epidemic* or occurrence or case or cases)).ab,kw,ti.	787

17	(((receiv* or recipient* or complet* or already or post or previously or after or following or "subsequent to" or since or had or despite or "in spite of") adj5 (immunis* or immuniz* or inoculat* or innoculat* or vaccin* or revaccinat* or reimmuniz* or reimmunis* or dose or doses) adj5 (infected or infection* or contracted or acquired or acquisition or caught or got* or transmi* or spread* or outbreak* or cluster* or communicab* or contagious* or epidemic* or occurrence or case or cases)) or ((infected or infection* or contracted or acquired or acquisition or caught or got* or transmi* or spread* or outbreak* or cluster* or communicab* or contagious* or epidemic* or occurrence or case or cases) adj5 (receiv* or recipient* or complet* or already or post or previously or after or following or "subsequent to" or since or had or despite or "in spite of") adj5 (immunis* or immuniz* or inoculat* or innoculat* or vaccin* or revaccinat* or reimmuniz* or reimmunis* or dose or doses))).ab,kw,ti.	24910
18	((vaccinated or immunized or immunised) adj5 (infected or infection* or contracted or acquired or acquisition or caught or got* or transmi* or spread* or outbreak* or cluster* or communicab* or contagious* or epidemic* or occurrence or case or cases)).ab,kw,ti.	9738
19	((vaccinated or immunized or immunised) adj10 (person or persons or people or man or men or woman or women or adult or adults or patient or patients or individual or individuals or responder or responders or practitioner* or staff or personnel or worker* or employee* or provider* or technician* or clinician* or doctor* or nurse or nurses or paramedic* or physician* or hospitalist* or pharmacist*) adj10 (infected or infection* or contracted or acquired or acquisition or caught or got* or transmi* or spread* or outbreak* or cluster* or communicab* or contagious* or epidemic* or occurrence or case or cases)).ab,kw,ti.	3761
20	((escape or escapes or escaped or escaping or evade or evades or evaded or evading or evasion) adj5 ("Ad26.COV2.S" or AZD1222 or BNT162* or ChAdOx1* or Comirnaty or immunis* or immuniz* or "JNJ-78436735" or "mrna-1273*" or Novavax or NVX-CoV2373 or PittCoVacc* or postvaccin* or postimmunis* or postimmuniz* or reimmunis* or reimmuniz* or revacciat* or tozinameran or vaccin*)).ab,kw,ti.	1213
21	13 or 14 or 15 or 16 or 17 or 18 or 19 or 20	182506
22	(wane or wanes or waned or waning).kw,ti.	578
23	((decay* or declin* or decreas* or impair* or duration or fail* or fall* or "go* down" or lack or lacks or lacked or lacking or lose or loses or losing or loss or lost or low or lower* or lesser* or poor or poorer* or "adversely affect*" or reduc*) adj5 (protect* or effectiv* or immunity or "immune respon*" or antibod* or sero*)).kw,ti. and (infected or infection* or contracted or acquired or acquisition or caught or got* or transmi* or spread* or outbreak* or cluster* or communicab* or contagious* or epidemic* or occurrence or case or cases).ab,kw,ti.	8094

24	((decay* or declin* or decreas* or impair* or duration or fail* or fall* or "go* down" or lack or lacks or lacked or lacking or lose or loses or losing or loss or lost or low or lower* or lesser* or poor or poorer* or "adversely affect*" or reduc*) adj5 (protect* or effectiv* or immunity or "immune respon*" or antibod* or sero*)).ab,kw,ti. and (infected or infection* or contracted or acquired or acquisition or caught or got* or transmi* or spread* or outbreak* or cluster* or communicab* or contagious* or epidemic* or occurrence or case or cases).kw,ti.	36564
25	22 or 23 or 24	42852
26	(1 or 6) and (12 or 21 or 25) and 9	700
27	4 and (12 or 25) and 9	962
28	26 or 27	1133
29	limit 28 to conference abstract	170
30	28 not 29	963
31	30 not (editorial or letter or note).pt.	934
32	limit 31 to yr="2021 -Current"	552
33	(202108* or 202109* or 202110* or 202111* or 202112* or 202201*).dd. or (summer 2021 or fall 2021 or autumn 2021 or winter 2021 or aug* 2021 or sep* 2021 or oct* 2021 or nov* 2021 or dec* 2021 or jan* 2022).dp.	647758
34	32 and 33	232
35	limit 34 to (english or french)	231

GLOBAL HEALTH

Global Health <1973 to 2021 Week 50>

	Searches	Results
1	((vaccines/ or immunization/ or vaccination/ or synthetic vaccines/ or combined vaccines/ or vaccine development/ or candidate vaccines/ or messenger RNA/) and (ncov or BNT162* or "mrna-1273").ti,ab,bt,hw,id,od,sh.) or (BNT162* or Comirnaty or CoronaVac or Covishield or Tozinameran or "mrna-1273*" or ChAdOx1* or AZD1222 or "Ad26.COV2.S" or "JNJ-78436735" or Novavax or NVX-CoV2373 or Ad5-nCoV or aAPC or "lentiviral minigene" or vaxzevria).ti,ab,bt,hw,id,od,sh.	910
2	("Severe acute respiratory syndrome coronavirus 2" or "coronavirus disease 2019" or "COVID-19" or "SARS-CoV-2").ti,ab,bt,hw,id,od,sh. or ("2019 corona virus" or "2019 coronavirus" or "2019 ncov" or "corona virus 19" or "corona virus 2019" or "corona virus disease 19" or "corona virus disease 2019" or "corona virus epidemic*" or "corona virus outbreak*" or "corona virus pandemic*" or "corona virus response" or "coronavirus 19" or "coronavirus 2019" or "coronavirus disease 19" or "coronavirus disease 2019" or "coronavirus epidemic*" or "coronavirus outbreak*" or "coronavirus pandemic*" or "coronavirus response" or "covid 19" or "covid 2019" or "new corona virus" or "new coronavirus" or "novel corona virus" or "novel coronavirus" or "novel	88811

	human coronavirus" or "sars coronavirus 2" or "sars cov 2" or "sars cov2" or "sars like coronavirus" or "severe acute respiratory syndrome corona virus 2" or "severe acute respiratory syndrome coronavirus 2" or "severe specific contagious pneumonia" or "wuhan corona virus" or "wuhan coronavirus" or ((pandemic* or novel or wuhan) adj3 (coronavirus* or "corona virus*" or betacoronavirus* or "beta coronavirus*" or "beta corona virus*" or pneumonia* or SARS or "severe acute respiratory syndrome")) or (pneumonia adj3 (coronavirus* or "corona virus*" or betacoronavirus* or "beta coronavirus*" or "beta corona virus*" or SARS or "severe acute respiratory syndrome")) or 2019ncov or covid or covid19 or covid2019 or ncov or sarscov2).ti,hw. or ("SARS-Cov-2 variant*" or VUI-202012-01 or "VOC-202012/01" or "B.1.1.7" or B117 or "B.1.351" or B1351 or "B.1.617" or B1617 or "P.1" or P1 or coronavirus* or "corona virus*" or betacoronavirus* or "beta coronavirus*" or "beta corona virus*" or pneumonia* or SARS or "severe acute respiratory syndrome").ti. or ((covid* or sars* or variant*) and (alpha or beta or gamma or delta or "variant* of concern" or "VOC")).ti. or ("2019 corona virus" or "2019 coronavirus" or "2019 ncov" or "corona virus 19" or "corona virus 2019" or "corona virus disease 19" or "corona virus disease 2019" or "corona virus epidemic*" or "corona virus outbreak*" or "corona virus pandemic*" or "corona virus response" or "coronavirus 19" or "coronavirus 2019" or "coronavirus disease 19" or "coronavirus disease 2019" or "coronavirus epidemic*" or "coronavirus outbreak*" or "coronavirus pandemic*" or "coronavirus response" or "covid 19" or "covid 2019" or "new corona virus" or "new coronavirus" or "novel corona virus" or "novel coronavirus" or "novel human coronavirus" or "sars coronavirus 2" or "sars cov 2" or "sars cov2" or "sars like coronavirus" or "severe acute respiratory syndrome corona virus 2" or "severe acute respiratory syndrome coronavirus 2" or "severe specific contagious pneumonia" or "wuhan corona virus" or "wuhan coronavirus" or ((pandemic* or novel or wuhan) adj3 (coronavirus* or "corona virus*" or betacoronavirus* or "beta coronavirus*" or "beta corona virus*" or pneumonia* or SARS or "severe acute respiratory syndrome")) or (pneumonia adj3 (coronavirus* or "corona virus*" or betacoronavirus* or "beta coronavirus*" or "beta corona virus*" or SARS or "severe acute respiratory syndrome")) or 2019ncov or covid or covid19 or covid2019 or ncov or sarscov2).ab. /freq=2	
3	(Omicron or "B.1.1.529" or "VOC-21NOV-01" or "B.1.1.529.1" or "B.1.1.529.2" or B11529*).ti,ab,bt,hw,id,od,sh.	0
4	2 or 3	88811
5	vaccines/ or immunization programmes/ or immunization/ or vaccination/ or passive immunization/ or synthetic vaccines/ or combined vaccines/ or vaccine development/ or candidate vaccines/ or (vaccin* or immunis* or immuniz* or innoculat* or inoculat* or jab* or shot* or injection* or nonimmunis* or nonimmuniz* or postvaccin* or postimmunis* or postimmuniz* or reimmunis* or reimmuniz* or revaccinat* or unimmunis* or unimmuniz* or ((one or single two or double or first or second or	282459

	partial* or full) adj2 (dose* or injection* or injected or injecting)) or single-dose* or double-dose* or BNT162* or Comirnaty or CoronaVac or Covishield or Tozinameran or "mrna-1273*" or ChAdOx1* or AZD1222 or "Ad26.COV2.S" or "JNJ-78436735" or Novavax or NVX-CoV2373 or Ad5-nCoV or aAPC or "lentiviral minigene" or vaxzevria).ti,ab,hw.	
6	4 and 5	10999
7	neutralizing antibodies/ or monoclonal antibodies/ or exp antibodies/ or antibody formation/ or antibody testing/ or antigen antibody reactions/ or humoral immunity/ or immune serum/ or immunity/ or immune response/ or immune system/ or cross immunity/ or cross reaction/ or passive immunity/ or immune competence/ or immunological factors/ or immunology/ or antibody dependent cellular cytotoxicity/ or lymphocytes/ or B lymphocytes/ or lymphocyte transformation/ or complement activation/ or Th2 lymphocytes/ or exp immunoglobulins/ or reinfection/ or viral load/ or seroconversion/ or seroprevalence/ or neutralization tests/ or virus neutralization/ or ELISA/ or (antibody or antibodies or antibody-positiv* or antibody-negativ* or "complement activation" or ((humoral or humoural or adaptive or antibody-mediated) adj3 (respon* or immun*)) or immunity or "immune system*" or "immune marker*" or ((immun* or sero*) adj3 respon*) or immunolog* or immunomodulat* or B-lymphocyte* or "B-cell*").ti,ab,bt,hw,id,od,sh. or (immun* or immunoglobulin* or IgA or IgD or IgE or IgG or IgM or sero* or sera*).ti,hw.	684729
8	("correlat* of protection" or "correlat* with protection" or "immune correlat*" or "correlat* of immunity" or "correlat* of neutraliz*" or "correlat* of neutralis*" or "measur* of protection" or "correlat* of infectivity" or "protective correlat*" or ((protective or protection) adj5 (level* or threshold* or marker* or limit* or amount* or mean* or baseline or quantif*)) or ((correlat* or marker* or threshold* or indicat* or measur*) adj5 (immun* or protect*)) or ((antibody or antibodies or titer* or titre*) adj3 (correlat* or threshold*)) or (protect* adj5 (immunity or antibod* or titer* or titre*)) or ((neutraliz* or neutralis*) adj2 (level* or amount* or limit* or mean* or baseline or quantif* or titer* or titre*)) or (predict* adj3 (immun* or protect*)) or "protective immunity" or "surrogate endpoint*").ti,ab,bt,hw,id,od,sh. or (protection or protective or protected or ((antibody or antibodies) adj2 (status or level*))).ti.	112171
9	7 and 8	72310
10	reinfection/ or ((viral load/ or asymptomatic infections/) and (re-infect* or reinfect*).ti,hw.)	2547
11	(((previous* or prior or past or post or postcovid* or post-covid* or postsars* or post-sars* or after or following or recover* or convalescen* or "after infection") adj3 infect*) or re-infect* or reinfect* or "after first infection").ti,ab,bt,hw,id,od,sh.	61095
12	10 or 11	61095
13	treatment failure/ or ((vaccin* or immunis* or immuniz*) adj3 fail*).ti,ab,bt,hw,id,od,sh.	10512

14	(((breakthrough* or "break through*") adj5 (vaccin* or infection*)) or "breakthrough rate*").ti,ab,bt,hw,id,od,sh.	771
15	(("second dose" or "two doses" or "fully vaccinated") adj5 (infected or infection* or contracted or acquired or acquisition or caught or got* or transmi* or spread* or outbreak* or cluster* or communicab* or contagious* or epidemic* or occurrence or case or cases)).ti,ab,bt,hw,id,od,sh.	220
16	((postvaccin* or postimmuniz* or postimmunis* or "post-vaccin*" or "post-immuniz*" or "post-immunis*") adj5 (infected or infection* or contracted or acquired or acquisition or caught or got* or transmi* or spread* or outbreak* or cluster* or communicab* or contagious* or epidemic* or occurrence or case or cases)).ti,ab,bt,hw,id,od,sh.	276
17	(((receiv* or recipient* or complet* or already or post or previously or after or following or "subsequent to" or since or had or despite or "in spite of") adj5 (immunis* or immuniz* or inoculat* or innoculat* or vaccin* or revaccinat* or reimmuniz* or reimmunis* or dose or doses) adj5 (infected or infection* or contracted or acquired or acquisition or caught or got* or transmi* or spread* or outbreak* or cluster* or communicab* or contagious* or epidemic* or occurrence or case or cases)) or ((infected or infection* or contracted or acquired or acquisition or caught or got* or transmi* or spread* or outbreak* or cluster* or communicab* or contagious* or epidemic* or occurrence or case or cases) adj5 (receiv* or recipient* or complet* or already or post or previously or after or following or "subsequent to" or since or had or despite or "in spite of") adj5 (immunis* or immuniz* or inoculat* or innoculat* or vaccin* or revaccinat* or reimmuniz* or reimmunis* or dose or doses))).ti,ab,bt,hw,id,od,sh.	8273
18	((vaccinated or immunized or immunised) adj5 (infected or infection* or contracted or acquired or acquisition or caught or got* or transmi* or spread* or outbreak* or cluster* or communicab* or contagious* or epidemic* or occurrence or case or cases)).ti,ab,bt,hw,id,od,sh.	4036
19	((vaccinated or immunized or immunised) adj10 (person or persons or people or man or men or woman or women or adult or adults or patient or patients or individual or individuals or responder or responders or practitioner* or staff or personnel or worker* or employee* or provider* or technician* or clinician* or doctor* or nurse or nurses or paramedic* or physician* or hospitalist* or pharmacist*) adj10 (infected or infection* or contracted or acquired or acquisition or caught or got* or transmi* or spread* or outbreak* or cluster* or communicab* or contagious* or epidemic* or occurrence or case or cases)).ti,ab,bt,hw,id,od,sh.	1732
20	((escape or escapes or escaped or escaping or evade or evades or evaded or evading or evasion) adj5 ("Ad26.COV2.S" or AZD1222 or BNT162* or ChAdOx1* or Comirnaty or immunis* or immuniz* or "JNJ-78436735" or "mrna-1273*" or Novavax or NVX-CoV2373 or PittCoVacc* or postvaccin* or postimmunis* or postimmuniz* or	427

	reimmunis* or reimmuniz* or revacciat* or tozinameran or vaccin*)).ti,ab,bt,hw,id,od,sh.	
21	13 or 14 or 15 or 16 or 17 or 18 or 19 or 20	23037
22	(wane or wanes or waned or waning).ti,bt,hw,id,od,sh.	122
23	((decay* or declin* or decreas* or impair* or duration or fail* or fall* or "go* down" or lack or lacks or lacked or lacking or lose or loses or losing or loss or lost or low or lower* or lesser* or poor or poorer* or "adversely affect*" or reduc*) adj5 (protect* or effectiv* or immunity or "immune respon*" or antibod* or sero*)).ti,hw. and (infected or infection* or contracted or acquired or acquisition or caught or got* or transmi* or spread* or outbreak* or cluster* or communicab* or contagious* or epidemic* or occurrence or case or cases).ti,ab,bt,hw,id,od,sh.	2939
24	((decay* or declin* or decreas* or impair* or duration or fail* or fall* or "go* down" or lack or lacks or lacked or lacking or lose or loses or losing or loss or lost or low or lower* or lesser* or poor or poorer* or "adversely affect*" or reduc*) adj5 (protect* or effectiv* or immunity or "immune respon*" or antibod* or sero*)).ti,ab,bt,hw,id,od,sh. and (infected or infection* or contracted or acquired or acquisition or caught or got* or transmi* or spread* or outbreak* or cluster* or communicab* or contagious* or epidemic* or occurrence or case or cases).ti,bt,hw,id,od,sh.	36265
25	22 or 23 or 24	36807
26	(1 or 6) and (12 or 21 or 25) and 9	428
27	4 and (12 or 25) and 9	677
28	26 or 27	747
29	limit 28 to yr="2021 -Current"	429
30	limit 29 to (english or french)	424

LITERATURE SEARCH

01/05/2022

COVID-19 antibody correlates of protection (revised): Supplementary Databases

Search update - 2022 Jan 05 (Part 2)

Request prepared by: Library Services
Contact information: library@oahpp.ca

Search results reporting

DATABASES SEARCHED

Database	Date searched	Records	Duplicates removed by database	Remaining
BIOSIS Previews	01/05/2022	368	0	368
SCOPUS	01/05/2022	866	0	866

RESULTS TOTALS

Records source	Records
Records identified through database searching	1,234
Duplicates removed by database	0
Duplicates removed by bibliographic management software	784
Total records after duplicates removed	150

Search strategies

BIOSIS

BIOSIS Previews <2021>

#	Searches	Results
1	(ncov or BNT162* or "mrna-1273" or "Ad5-nCoV vaccine" or "BNT162 vaccine" or "ChAdOx1 COVID-19 vaccine" or "Covid-19 aAPC vaccine" or "lentiviral minigene vaccine of COVID-19 coronavirus" or "mRNA-1273 vaccine" or "recombinant SARS-CoV-2 vaccine NVX-cov2373" or "SARS-CoV-2 inactivated vaccines").rn. or (BNT162* or Comirnaty or CoronaVac or Covishield or Tozinameran or "mrna-1273*" or ChAdOx1* or AZD1222 or "Ad26.COV2.S" or "JNJ-78436735" or Novavax or NVX-CoV2373 or Ad5-nCoV or aAPC or "lentiviral minigene" or vaxzevria).ti,tw.	715
2	("Severe acute respiratory syndrome coronavirus 2" or "coronavirus disease 2019" or "COVID-19" or "SARS-CoV-2" or ("2019 corona virus" or "2019 coronavirus" or "2019 ncov" or "corona virus 19" or "corona virus 2019" or "corona virus disease 19" or "corona virus disease 2019" or "corona virus epidemic*" or "corona virus outbreak*" or "corona virus pandemic*" or "corona virus response" or "coronavirus 19" or	53052

	"coronavirus 2019" or "coronavirus disease 19" or "coronavirus disease 2019" or "coronavirus epidemic*" or "coronavirus outbreak*" or "coronavirus pandemic*" or "coronavirus response" or "covid 19" or "covid 2019" or "new corona virus" or "new coronavirus" or "novel corona virus" or "novel coronavirus" or "novel human coronavirus" or "sars coronavirus 2" or "sars cov 2" or "sars cov2" or "sars like coronavirus" or "severe acute respiratory syndrome corona virus 2" or "severe acute respiratory syndrome coronavirus 2" or "severe specific contagious pneumonia" or "wuhan corona virus" or "wuhan coronavirus" or ((pandemic* or novel or wuhan) adj3 (coronavirus* or "corona virus*" or betacoronavirus* or "beta coronavirus*" or "beta corona virus*" or pneumonia* or SARS or "severe acute respiratory syndrome")) or (pneumonia adj3 (coronavirus* or "corona virus*" or betacoronavirus* or "beta coronavirus*" or "beta corona virus*" or SARS or "severe acute respiratory syndrome")) or 2019ncov or covid or covid19 or covid2019 or ncov or sarscov2)).ti,hw,mc. or ("SARS-Cov-2 variant*" or VUI-202012-01 or "VOC-202012/01" or "B.1.1.7" or B117 or "B.1.351" or B1351 or "B.1.617" or B1617 or "P.1" or P1 or coronavirus* or "corona virus*" or betacoronavirus* or "beta coronavirus*" or "beta corona virus*" or pneumonia* or SARS or "severe acute respiratory syndrome").ti. or ((covid* or sars* or variant*) and (alpha or beta or gamma or delta or "variant* of concern" or "VOC")).ti. or ("2019 corona virus" or "2019 coronavirus" or "2019 ncov" or "corona virus 19" or "corona virus 2019" or "corona virus disease 19" or "corona virus disease 2019" or "corona virus epidemic*" or "corona virus outbreak*" or "corona virus pandemic*" or "corona virus response" or "coronavirus 19" or "coronavirus 2019" or "coronavirus disease 19" or "coronavirus disease 2019" or "coronavirus epidemic*" or "coronavirus outbreak*" or "coronavirus pandemic*" or "coronavirus response" or "covid 19" or "covid 2019" or "new corona virus" or "new coronavirus" or "novel corona virus" or "novel coronavirus" or "novel human coronavirus" or "sars coronavirus 2" or "sars cov 2" or "sars cov2" or "sars like coronavirus" or "severe acute respiratory syndrome corona virus 2" or "severe acute respiratory syndrome coronavirus 2" or "severe specific contagious pneumonia" or "wuhan corona virus" or "wuhan coronavirus" or ((pandemic* or novel or wuhan) adj3 (coronavirus* or "corona virus*" or betacoronavirus* or "beta coronavirus*" or "beta corona virus*" or pneumonia* or SARS or "severe acute respiratory syndrome")) or (pneumonia adj3 (coronavirus* or "corona virus*" or betacoronavirus* or "beta coronavirus*" or "beta corona virus*" or SARS or "severe acute respiratory syndrome")) or 2019ncov or covid or covid19 or covid2019 or ncov or sarscov2).ab. /freq=2	
3	(Omicron or "B.1.1.529" or "VOC-21NOV-01" or "B.1.1.529.1" or "B.1.1.529.2" or B11529*).ab,ti,kw,hw,tw,mc.	50
4	2 or 3	53100
5	(vaccin* or immunis* or immuniz* or innoculat* or inoculat* or jab* or shot* or injection* or nonimmunis* or nonimmuniz* or postvaccin* or postimmunis* or	90677

	postimmuniz* or reimmunis* or reimmuniz* or revaccinat* or unimmunis* or unimmuniz* or ((one or single two or double or first or second or partial* or full) adj2 (dose* or injection* or injected or injecting)) or single-dose* or double-dose* or BNT162* or Comirnaty or CoronaVac or Covishield or Tozinameran or "mrna-1273*" or ChAdOx1* or AZD1222 or "Ad26.COV2.S" or "JNJ-78436735" or Novavax or NVX-CoV2373 or Ad5-nCoV or aAPC or "lentiviral minigene" or vaxzevria).ti,ab,hw,tw,mc.	
6	4 and 5	8270
7	(antibody or antibodies or antibody-positiv* or antibody-negativ* or "complement activation" or ((humoral or humoural or adaptive or antibody-mediated) adj3 (respon* or immun*)) or immunity or "immune system*" or "immune marker*" or ((immun* or sero*) adj3 respon*) or immunolog* or immunomodulat* or B-lymphocyte* or "B-cell*").ti,ab,hw,tw,mc. or (immun* or immunoglobulin* or IgA or IgD or IgE or IgG or IgM or sero* or sera*).ti,hw.	373129
8	("correlat* of protection" or "correlat* with protection" or "immune correlat*" or "correlat* of immunity" or "correlat* of neutraliz*" or "correlat* of neutralis*" or "measur* of protection" or "correlat* of infectivity" or "protective correlat*" or ((protective or protection) adj5 (level* or threshold* or marker* or limit* or amount* or mean* or baseline or quantif*)) or ((correlat* or marker* or threshold* or indicat* or measur*) adj5 (immun* or protect*)) or ((antibody or antibodies or titer* or titre*) adj3 (correlat* or threshold*)) or (protect* adj5 (immunity or antibod* or titer* or titre*)) or ((neutraliz* or neutralis*) adj2 (level* or amount* or limit* or mean* or baseline or quantif* or titer* or titre*)) or (predict* adj3 (immun* or protect*)) or "protective immunity" or "surrogate endpoint*").ti,ab,hw,tw,mc. or (protection or protective or protected or ((antibody or antibodies) adj2 (status or level*))).ti.	33075
9	7 and 8	20510
10	(((previous* or prior or past or post or postcovid* or post-covid* or postsars* or post-sars* or after or following or recover* or convalescen* or "after infection") adj3 infect*) or re-infect* or reinfect* or "after first infection").ti,ab,hw,tw,mc.	13256
11	((vaccin* or immunis* or immuniz*) adj3 fail*).ti,ab,hw,tw,mc.	303
12	(((breakthrough* or "break through*") adj5 (vaccin* or infection*)) or "breakthrough rate*").ti,ab,hw,tw,mc.	181
13	(("second dose" or "two doses" or "fully vaccinated") adj5 (infected or infection* or contracted or acquired or acquisition or caught or got* or transmi* or spread* or outbreak* or cluster* or communicab* or contagious* or epidemic* or occurrence or case or cases)).ti,ab,hw,tw,mc.	58
14	((postvaccin* or postimmuniz* or postimmunis* or "post-vaccin*" or "post-immuniz*" or "post-immunis*") adj5 (infected or infection* or contracted or acquired or acquisition or caught or got* or transmi* or spread* or outbreak* or cluster* or communicab* or contagious* or epidemic* or occurrence or case or cases)).ti,ab,hw,tw,mc.	89

15	(((receiv* or recipient* or complet* or already or post or previously or after or following or "subsequent to" or since or had or despite or "in spite of") adj5 (immunis* or immuniz* or inoculat* or innoculat* or vaccin* or revaccinat* or reimmuniz* or reimmunis* or dose or doses) adj5 (infected or infection* or contracted or acquired or acquisition or caught or got* or transmi* or spread* or outbreak* or cluster* or communicab* or contagious* or epidemic* or occurrence or case or cases)) or ((infected or infection* or contracted or acquired or acquisition or caught or got* or transmi* or spread* or outbreak* or cluster* or communicab* or contagious* or epidemic* or occurrence or case or cases) adj5 (receiv* or recipient* or complet* or already or post or previously or after or following or "subsequent to" or since or had or despite or "in spite of") adj5 (immunis* or immuniz* or inoculat* or innoculat* or vaccin* or revaccinat* or reimmuniz* or reimmunis* or dose or doses))).ti,ab,hw,tw,mc.	1595
16	((vaccinated or immunized or immunised) adj5 (infected or infection* or contracted or acquired or acquisition or caught or got* or transmi* or spread* or outbreak* or cluster* or communicab* or contagious* or epidemic* or occurrence or case or cases)).ti,ab,hw,tw,mc.	720
17	((vaccinated or immunized or immunised) adj10 (person or persons or people or man or men or woman or women or adult or adults or patient or patients or individual or individuals or responder or responders or practitioner* or staff or personnel or worker* or employee* or provider* or technician* or clinician* or doctor* or nurse or nurses or paramedic* or physician* or hospitalist* or pharmacist*) adj10 (infected or infection* or contracted or acquired or acquisition or caught or got* or transmi* or spread* or outbreak* or cluster* or communicab* or contagious* or epidemic* or occurrence or case or cases)).ti,ab,hw,tw,mc.	282
18	((escape or escapes or escaped or escaping or evade or evades or evaded or evading or evasion) adj5 ("Ad26.COV2.S" or AZD1222 or BNT162* or ChAdOx1* or Comirnaty or immunis* or immuniz* or "JNJ-78436735" or "mrna-1273*" or Novavax or NVX-CoV2373 or PittCoVacc* or postvaccin* or postimmunis* or postimmuniz* or reimmunis* or reimmuniz* or revacciat* or tozinameran or vaccin*)).ti,ab,hw,tw,mc.	180
19	10 or 11 or 12 or 13 or 14 or 15 or 16 or 17 or 18	15363
20	(wane or wanes or waned or waning).ti,hw,tw.	627
21	((decay* or declin* or decreas* or impair* or duration or fail* or fall* or "go* down" or lack or lacks or lacked or lacking or lose or loses or losing or loss or lost or low or lower* or lesser* or poor or poorer* or "adversely affect*" or reduc*) adj5 (protect* or effectiv* or immunity or "immune respon*" or antibod* or sero*)).ti,hw,tw. and (infected or infection* or contracted or acquired or acquisition or caught or got* or transmi* or spread* or outbreak* or cluster* or communicab* or contagious* or epidemic* or occurrence or case or cases).ti,ab,hw,tw,mc.	12710

22	((decay* or declin* or decreas* or impair* or duration or fail* or fall* or "go* down" or lack or lacks or lacked or lacking or lose or loses or losing or loss or lost or low or lower* or lesser* or poor or poorer* or "adversely affect*" or reduc*) adj5 (protect* or effectiv* or immunity or "immune respon*" or antibod* or sero*)).ti,ab,hw,tw,mc. and (infected or infection* or contracted or acquired or acquisition or caught or got* or transmi* or spread* or outbreak* or cluster* or communicab* or contagious* or epidemic* or occurrence or case or cases).hw,ti,tw.	12710
23	20 or 21 or 22	13204
24	(1 or 6) and (19 or 23) and 9	295
25	4 and (10 or 23) and 9	475
26	24 or 25	502
27	limit 26 to yr="2021 -Current"	370
28	limit 27 to (english or french)	368

SCOPUS

#	Queries	Results
#1	TITLE-ABS ("Ad5-nCoV vaccine" OR "BNT162 vaccine" OR "ChAdOx1 COVID-19 vaccine" OR "Covid-19 aAPC vaccine" OR "lentiviral minigene vaccine of COVID-19 cORonavirus" OR "mRNA-1273 vaccine" OR "recombinant SARS-CoV-2 vaccine NVX-cov2373" OR "SARS-CoV-2 inactivated vaccines") OR TITLE-ABS (bnt162* OR comirnaty OR coronavac OR covishield OR tozinameran OR "mrna-1273*" OR chadox1* OR azd1222 OR "Ad26.COV2.S" OR "JNJ-78436735" OR novavax OR nvx-cov2373 OR ad5-ncov OR aapc OR "lentiviral minigene" OR vaxzevria)	2,721
#2	(TITLE-ABS ("Severe acute respiratory syndrome coronavirus 2" OR "coronavirus disease 2019" OR "COVID-19" OR "SARS-CoV-2")) OR (TITLE-ABS ("2019 corona virus" OR "2019 coronavirus" OR "2019 ncov" OR "corona virus 19" OR "corona virus 2019" OR "corona virus disease 19" OR "corona virus disease 2019" OR "corona virus epidemic*" OR "corona virus outbreak*" OR "corona virus pandemic*" OR "corona virus response" OR "coronavirus 19" OR "coronavirus 2019" OR "coronavirus disease 19" OR "coronavirus disease 2019" OR "coronavirus epidemic*" OR "coronavirus outbreak*" OR "coronavirus pandemic*" OR "coronavirus response" OR "covid 19" OR "covid 2019" OR "new corona virus" OR "new coronavirus" OR "novel corona virus" OR "novel coronavirus" OR "novel human coronavirus" OR "sars coronavirus 2" OR "sars cov 2" OR "sars cov2" OR "sars like coronavirus" OR "severe acute respiratory syndrome corona virus 2" OR "severe acute respiratory syndrome coronavirus 2" OR "severe specific contagious pneumonia" OR "wuhan corona virus" OR "wuhan coronavirus" OR ((pandemic* OR novel OR wuhan) W/3 (coronavirus* OR "corona virus*" OR betacoronavirus* OR "beta coronavirus*" OR "beta corona virus*" OR pneumonia* OR sars OR "severe acute respiratory syndrome")) OR (pneumonia W/3 (coronavirus* OR "corona virus*" OR betacoronavirus* OR "beta coronavirus*" OR "beta corona virus*" OR sars OR "severe acute respiratory syndrome")) OR 2019ncov OR covid OR covid19 OR covid2019 OR ncov OR sarscov2)) OR (TITLE ("SARS-Cov-2 variant*" OR vui-202012-01 OR "VOC-202012/01" OR "B.1.1.7" OR b117 OR "B.1.351" OR b1351 OR "B.1.617" OR b1617 OR "P.1" OR p1 OR coronavirus* OR "corona virus*" OR	405, 697

	betacoronavirus* OR "beta coronavirus*" OR "beta corona virus*" OR pneumonia* OR sars OR "severe acute respiratory syndrome") OR TITLE ((covid* OR sars* OR variant*) AND (alpha OR beta OR gamma OR delta OR "variant* of concern" OR "VOC")))	
#3	TITLE-ABS-KEY (omicron OR "B.1.1.529" OR "VOC-21NOV-01" OR "B.1.1.529.1" OR "B.1.1.529.2" OR b11529*)	1,745
#4	#2 OR #3	407,402
#5	TITLE-ABS ((vaccin* OR immunis* OR immuniz* OR innoculat* OR inoculat* OR jab* OR shot* OR injection* OR nonimmunis* OR nonimmuniz* OR postvaccin* OR postimmunis* OR postimmuniz* OR reimmunis* OR reimmuniz* OR revaccinat* OR unimmunis* OR unimmuniz* OR ((one OR single OR two OR double OR first OR second OR partial* OR full) W/2 (dose* OR injection* OR injected OR injecting)) OR single-dose* OR double-dose* OR bnt162* OR comirnaty OR coronavac OR covishield OR tozinameran OR "mrna-1273*" OR chadox1* OR azd1222 OR "Ad26.COV2.S" OR "JNJ-78436735" OR novavax OR nvx-cov2373 OR ad5-ncov OR aapc OR "lentiviral minigene" OR vaxzevria))	2,043,780
#6	#4 AND #5	33,299
#7	TITLE-ABS (antibody OR antibodies OR antibody-positiv* OR antibody-negativ* OR "complement activation" OR ((humoral OR humoural OR adaptive OR antibody-mediated) W/3 (respon* OR immun*)) OR immunity OR "immune system*" OR "immune marker*" OR ((immun* OR sero*) W/3 respon*) OR immunolog* OR immunomodulat* OR b-lymphocyte* OR "B-cell*") OR TITLE (immun* OR immunoglobulin* OR iga OR igd OR ige OR igg OR igm OR sero* OR sera*)	2,474,238
#8	TITLE-ABS ("correlat* of protection" OR "correlat* with protection" OR "immune correlat*" OR "correlat* of immunity" OR "correlat* of neutraliz*" OR "correlat* of neutralis*" OR "measur* of protection" OR "correlat* of infectivity" OR "protective correlat*" OR ((protective OR protection) W/5 (level* OR threshold* OR marker* OR limit* OR amount* OR mean* OR baseline OR quantif*)) OR ((correlat* OR marker* OR threshold* OR indicat* OR measur*) W/5 (immun* OR protect*)) OR ((antibody OR antibodies OR titer* OR titre*) W/3 (correlat* OR threshold*)) OR (protect* W/5 (immunity OR antibod* OR titer* OR titre*)) OR ((neutraliz* OR neutralis*) W/2 (level* OR amount* OR limit* OR mean* OR baseline OR quantif* OR titer* OR titre*)) OR (predict* W/3 (immun* OR protect*)) OR "protective immunity" OR "surrogate endpoint*") OR TITLE (protection OR protective OR protected OR ((antibody OR antibodies) W/2 (status OR level*)))	693,727
#9	#7 AND #8	205,010
#10	(#1 OR #6) AND #9	2,480
#11	#4 AND #9	4,810
#12	#10 OR #11	4,837
#13	LANGUAGE(English) OR LANGUAGE(French) AND PUBYEAR > 2020	3,609,628
#14	#12 AND #13	2,389
#15	#14 AND NOT INDEX (MEDLINE)	866
