# Supplementary material for: Does a humoral correlate of protection exist for SARS-CoV-2? A systematic review
Source: PLoS One. 2022 Apr 8;17(4):e0266852. doi: 10.1371/journal.pone.0266852 (PMC8993021; doi:10.1371/journal.pone.0266852)
Supplement: S3 Table — (ZIP) [file pone.0266852.s003.zip › Case reports 2022_01_28.pdf]

Quality Assessment- Case reports (Page 1 left)

Author (year)	Q1. Was the study question or objective clearly stated?	Q1. Free-text field	Q2. Was the study population clearly and fully described, including a case definition?	Q2. Free-text field	Q3. Were the cases consecutive?	Q3. Free-text field	Q4. Were the subjects comparable?	Q4. Free-text field	Q5. Was the intervention clearly described?	Q5. Free-text field	Q6. Were the outcome measures clearly defined, valid, reliable, and implemented consistently across all study participants?	Q6. Free-text field	Q7. Was the length of follow-up adequate?	Q7. Free-text field	Q8. Were the statistical methods well-described?	Q8. Free-text field	Q9. Were the results well-described?
Roy (2021)	Yes		Yes		Other (specify in free text column)	single case	Other (specify in free text column)	single case	Yes		Yes		Yes		Other (specify in free text column)	not applicable; single case report	Yes
Strafella (2021)	No		Yes		Other (specify in free text column)	single case	Other (specify in free text column)	single case	Yes	vaccination with Pfizer	Yes		Yes		Yes	evaluation of viral load decay coompared to reference group	Yes
Ul-Haq (2020)	No		Yes		Other (specify in free text column)	single case	Other (specify in free text column)	single case	Yes	first infection described clinically and serologically	No	no description of assay, no antibody units given	Yes		Other (specify in free text column)	not applicable; single case report	No
Vetter (2021)	Yes		Yes		Other (specify in free text column)	single case	Other (specify in free text column)	single case	Yes	both clinical and serological description of first infection	Yes		Yes		Other (specify in free text column)	not applicable; single case report	Yes
Brehm (2021)	Yes		Yes		Other (specify in free text column)	not applicable	Other (specify in free text column)	single case	Yes	extensive description of both infections	Yes		Yes		Other (specify in free text column)	not applicable; single case report	Yes
Inada (2021)	Yes		Yes		Other (specify in free text column)	single case in a household cluster	Other (specify in free text column)	single case in a household cluster	Yes	good description of first infection	Yes		Yes		Other (specify in free text column)	not applicable; single case report	Yes
Schulte (2021)	Yes		Yes		Yes		Yes	members of a household with likely similar exposure patterns, but differing demographics	Yes		Yes		Yes		Other (specify in free text column)	not applicable	Yes
Selhorst (2020)	Yes		Yes		Yes		Other (specify in free text column)	3 patients, 1 healthcare worker	Yes		Yes		Yes		Other (specify in free text column)	not applicable	Yes
Munivenkatappa (2021)	Yes		Yes		Other (specify in free text column)	single case report	Other (specify in free text column)	single case	Yes		Yes		Yes		Other (specify in free text column)	not applicable; single case report	Yes
Kohler (2021)	Yes		Yes		Yes		Yes		Yes		Yes		Yes		Other (specify in free text)	not applicable	Yes

Quality Assessment- Case reports (Page 1 right)

Q9. Free-text field			Q.15 Were titres reported in the manuscript taken within 1 month of re-infection or vaccine breakthrough?	Q16. Were antibodies measured at peak (30-60 days from first infection or vaccination)?	Q17. Were re-infections/breatkthrough infections documented to be due to a VOC?	Q10. Was the paper high enough quality to be used in the review?	Q10. Free-text field
			No	No	Not reported	Yes	
			Yes	Yes	Yes; B.117	Yes	
lack of detail regarding serological assay and units			No	No	Not reported	Yes	
very thorough timeline of infection and serological measures			No	Yes	Not reported	Yes	
			No	No	Yes; initial infection with B.3, reinfection with B.1.177	Yes	
			Yes	No	Not reported	Yes	
			No	No	B.1.525	Yes	
			No	No	G clade	Yes	
	Yes	No	No	Yes			
	No	No	Not documented	Yes		No	
