# Supplementary material for: Does a humoral correlate of protection exist for SARS-CoV-2? A systematic review
Source: PLoS One. 2022 Apr 8;17(4):e0266852. doi: 10.1371/journal.pone.0266852 (PMC8993021; doi:10.1371/journal.pone.0266852)
Supplement: S3 Table — (ZIP) [file pone.0266852.s003.zip › QA case control 2022_01_28.pdf]

Quality Assessment- Case-control studies (Page 1 left)

Author (year)	Q1. Was the research question or objective in this paper clearly stated and appropriate?	Q1. Free-text field	Q2. Was the study population clearly specified and defined?	Q2. Free-text field	Q3. Did the authors include a sample size justification?	Q3. Free-text field	Q4. Were controls selected or recruited from the same or similar population that gave rise to the cases (including the same timeframe)?	Q4. Free-text field	Q5. Were the definitions, inclusion and exclusion criteria, algorithms or processes used to identify or select cases and controls valid, reliable, and implemented consistently across all study participants?	Q5. Free-text field	Q6. Were the cases clearly defined and differentiated from controls?	Q6. Free-text field	Q7. If less than 100 percent of eligible cases and/or controls were selected for the study, were the cases and/or controls randomly selected from those eligible?	Q7. Free-text field	Q8. Was there use of concurrent controls?	Q8. Free-text field	Q9. Were the investigators able to confirm that the exposure/risk occurred prior to the development of the condition or event that defined a participant as a case?	Q9. Free-text field
Bergwerk (2021)	Yes		Yes		Other (specify in free text column)	not applicable, all breakthrough cases for which serology was available were included	Yes		Yes		Yes		Yes		Yes		Yes	
Gilbert (2021)	Yes		Yes		Yes		Yes		Yes		Yes		Yes		Yes		Yes	
Yamamoto (2021)	Yes		Yes		No		Yes		Yes		Yes		Not applicable	all seropositive cases were followed for breakthrough infection	Yes		Yes	

Quality Assessment- Case-control studies (Page 1 right)

Q10. Were the measures of exposure/risk clearly defined, valid, reliable, and implemented consistently (including the same time period) across all study participants?	Q10. Free-text field	Q11. Were the assessors of exposure/risk blinded to the case or control status of participants?	Q11. Free-text field	Q12. Were key potential confounding variables measured and adjusted statistically in the analyses? If matching was used, did the investigators account for matching during study analysis?	Q12. Free-text field	Q.13 Were titres reported in the manuscript taken within 1 month of re-infection or vaccine breakthrough?	Q14. Were antibodies measured at peak (30-60 days from first infection or vaccination)?	Q15. Were re-infections/breatkthrough infections documented to be due to a VOC?	Q16. Was the paper high enough quality to be used in the review?	Q16. Free-text field
Yes		Other (specify in free text column)	not reported	Yes		Yes	Yes	28/33 breakthroughs B.117	Yes	Both peak and peri-infection titres reported, and ratios derived between cases/controls for each
Yes		Other (specify in free text column)	not reported	Yes		N/A	Yes	N/A	Yes	
Yes		Other (specify in free text column)	not reported	Yes		No	Yes	5/17 due to Delta, rest not sequenced	Yes	
