# Supplementary material for: Does a humoral correlate of protection exist for SARS-CoV-2? A systematic review
Source: PLoS One. 2022 Apr 8;17(4):e0266852. doi: 10.1371/journal.pone.0266852 (PMC8993021; doi:10.1371/journal.pone.0266852)
Supplement: S3 Table — (ZIP) [file pone.0266852.s003.zip › QA_Cohort 2022_01_28.pdf]

Quality Assessment- Cohort Studies (Page 1 left)

Author (year)	Q1. Was the research question or objective in this paper clearly stated?	Q1. Free-text field	Q2. Was the study population clearly specified and defined?	Q2. Free-text field	Q3. Was the participation rate of eligible persons at least 50%?	Q3. Free-text field	Q4. Were all the subjects selected or recruited from the same or similar populations (including the same time period)? Were inclusion and exclusion criteria for being in the study prespecified and applied uniformly to all participants?	Q4. Free-text field	Q5. Was a sample size justification, power description, or variance and effect estimates provided?	Q5. Free-text field	Q6. For the analyses in this paper, were the exposure(s) of interest measured prior to the outcome(s) being measured?	Q6. Free-text field	Q7. Was the timeframe sufficient so that one could reasonably expect to see an association between exposure and outcome if it existed?	Q7. Free-text field	Q8. For exposures that can vary in amount or level, did the study examine different levels of the exposure as related to the outcome (e.g., categories of exposure, or exposure measured as continuous variable)?	Q8. Free-text field	Q9. Were the exposure measures (independent variables) clearly defined, valid, reliable, and implemented consistently across all study participants?	Q9. Free-text field
Dimeglio (2021)	No		No	Study population stated to be French HCW, but no further population characteristics given	Yes		Yes		Other (specify in free text column)	not applicable, broad serosurvey of eligible HCW	Yes		Yes		No	antibody titres for breakthrough reported, antibody levels of protected not reported	Yes	
Krutikov (2021)	Yes		Yes		Yes		No	Both staff and LTC residents were recruited, assessed separately by excluding staff older than 65 years and residents younger than 65 years to avoid misclassification	Other (specify in free text column)	not applicable, broad serosurvey of eligible HCW and LTC residents	Yes		Yes		Yes		Yes	
Leidi (2021)	Yes		Yes		No	a representative random sample of adults living in Geneva was used	Yes	broad study population (healthy adults in a Swiss region)	Yes		Yes		Yes		Yes	both seropositive and seronegative at baseline evaluated over time	Yes	
Lumley (2021)	Yes		Yes		Yes		Yes	health care workers at the same facility	Other (specify in free text column)	not applicable, broad longitudinal serosurvey of workers	Yes		Yes		Yes	both seropositive and seronegative at baseline evaluated over time	Yes	
Ali (2021)	No		No		No	serosurvey of 829 patients admitted to hospital during a specific timeframe	No	broad age range (10-60 years), no information given regarding reason for hospitalization or comorbidities	Other (specify in free text column)	not applicable, large serosurvey	Yes		Yes		Yes		No	only one of 26 patients has reported pre-re-exposure serology reported
Gallais (2021)	Yes		Yes		No	large serosurvey of HCW	Yes		Other (specify in free text column)	not applicable, large serosurvey	Yes		Yes		Yes		Yes	
Wilkins (2021)	Yes		Yes		Other (specify in free text column)	unknown; large study population, but no total population reported	Yes		Other (specify in free text column)	not applicable, large serosurvey	Yes		Yes		Yes		Yes	
Michos (2021)	Yes		Yes		No	small sample size of 268 HCW from a large hospital	Yes	HCWs at the same hospital	No		Yes		Yes		Yes	titres assessed after both 1st and 2nd dose of BNT162b2	Yes	

Quality Assessment- Cohort Studies (Page 2 left)

Brunner (2021)	Yes	Yes		Yes		Yes	HCWs at the same hospital	No	No	Antibody titre reported is "in between first vaccination and positive PCR result", and does not represent the titre for double vaccinees prior to infection	Yes	No	no titre reported following second vaccine dose	Yes	
Blain (2021)	Yes	Yes		Yes		Yes	residents of LTC	No	Yes		Yes	Yes		No	temporality of serology unclear; NH1 residents had outbreak 3-5 months apart, NH2 had 5-6 month between outbreaks, all grouped together in Table 2
Khoury (2021)	Yes	Yes		Other (specify in free text column)	Secondary analysis of clinical trial data, no information regarding participation rates was provided	Yes		No	Yes		Yes	No		Yes	
Earle (2021)	Yes	Yes		Other (specify in free text column)	Secondary analysis of clinical trial data, no information regarding participation rates was provided	Yes		No	Yes		Yes	No		Yes	
Cromer (2021)	Yes	Yes		Other (specify in free text column)	Secondary analysis of clinical trial data, no information regarding participation rates was provided	Yes		No	Yes		Yes	No		Yes	
Goldblatt (2021)	Yes	Yes		Other (specify in free text column)	Secondary analysis of clinical trial data, no information regarding participation rates was provided	Yes		No	Yes		Yes	Yes		Yes	
Feng (2021)	Yes	Yes		Other (specify in free text column)	Secondary analysis of clinical trial data, no information regarding participation rates was provided	Yes		No	Yes		Yes	No		Yes	
Cohen (2021)	Yes	Yes		Other (specify in free text column)	Secondary analysis of clinical trial data, no information regarding participation rates was provided	Yes		No	Yes		Yes	No		Yes	
Mlocochova (2021)	No	No	study population is vaguely defined as "new cases of SARS-CoV-2 in India since the start of 2021".	Other (specify in free text column)	unknown; no sample size given	No	"new cases of SARS-CoV-2 in India"	No	No	Unclear when sampling was performed, appears to be post-infection	Yes	Yes		No	unclear sample definition, timepoints of serology
Maier (2021)	Yes	Yes		Other (specify in free text column)	Sub-study examining COVID infections in a larger cohort	No	all age ranges (0-9 years reported separately from 10+ years)	No	Yes		Yes	Yes		Yes	
Pierobon (2021)	Yes	Yes		Other (specify in free text column)	Sub-study examining COVID infections in a larger cohort	No	LTC residents and HC workers	No	Other	Unknown (titres measured "at the beginning of the outbreak"	Yes	Yes		No	Unclear when Ab measurements were taken relative to infection for residents other than the index case

Quality Assessment- Cohort Studies (Page 3 left)

Aldridge (2021)	Yes	Yes	Yes	Not calculated in paper, but they had a cohort of 10,330 people and 8858 individuals (86%)	Yes	No	Yes	Yes	Yes	Yes

Quality Assessment- Cohort Studies (Page 1 right)

Q10. Was the exposure(s) assessed more than once over time?	Q10. Free-text field	Q11. Were the outcome measures (dependent variables) clearly defined, valid, reliable, and implemented consistently across all study participants?	Q11. Free-text field	Q12. Were the outcome assessors blinded to the exposure status of participants?	Q12. Free-text field	Q13. Was loss to follow-up after baseline 20% or less?	Q13. Free-text field	Q14. Were key potential confounding variables measured and adjusted statistically for their impact on the relationship between exposure(s) and outcome(s)?	Q14. Free-text field	Q.15 Were titres reported in the manuscript taken within 1 month of re-infection or vaccine breakthrough?	Q16. Were antibodies measured at peak (30-60 days from first infection or vaccination)?	Q17. Were re-infections/breatkthrough infections documented to be due to a VOC?	Q18. Was the paper high enough quality to be used in the review?	Q15. Free-text field
Yes		No	no clear definition of re-infection given in the paper, although information contained therein meets our definition	No	breakthrough/re-infections identified for analysis	Other (specify in free text column)	loss to follow-up not reported	No		Not reported	Not reported	Not reported	Yes	
Yes		Yes		No	breakthrough/re-infections identified for analysis	No		Yes		Some	Not reported	Not reported	Yes	Study reports high anti-S and anti-N patients within a month of re-infection for a small number of participants, however no titres reported for protected individuals for comparison
Yes		Yes		No	breakthrough/re-infections identified for analysis	Other (specify in free text column)	not reported	Yes		Yes, in 1 of 5 cases of re-infection	Not reported	Not reported	Yes	
Yes		Yes		No	breakthrough/re-infections identified for analysis	Other (specify in free text column)	not reported	Yes		Yes, in only 1 instance	No	Not reported	Yes	
Yes		No	no clear definition of re-infection given in the paper, although information contained therein meets our definition for some (not all) study participants	No	breakthrough/re-infections identified for analysis	Other (specify in free text column)	not reported	No	no adjustment for demographics, time since exposure and outcome etc.	Not reported	No	Not reported	Yes	
Yes		Yes		No	breakthrough/re-infections identified for analysis	Other (specify in free text column)	not reported	Yes		No	No	Not reported	Yes	
Yes		Yes		No	breakthrough/re-infections identified for analysis	No	Baseline enrollment 6510, follow-up testing on 4947 (loss of 24%)	Yes		No	Not reported	Not reported	Yes	
Yes		Yes		No	breakthrough/re-infections identified for analysis	No		Yes		Yes	Yes	Not reported	Yes	

Quality Assessment- Cohort Studies (Page 2 right)

No	Yes		No	breakthrough/re-infections identified for analysis	No		Yes		Not reported	Not reported	B.117 (9 cases), B.1351 (2 cases), B.1617.2 (4 cases)	No	Titres reported following first doses only, such that an evaluation of most recent titre for double vaccine recipients is impossible
No	Yes		No	breakthrough/re-infections identified for analysis	No	evaluable outcomes in 55.6% of sample	Yes		N/A	Yes	B.1.617.2	No	Difficulty determining the temporality of vaccination; incomplete discription of data included in each category in Table 2.
No	Yes		No		Other (specify in free text column)	Cross-sectional analysis	No		N/A	N/A	N/A	Yes	
No	Yes		No		Other (specify in free text column)	Cross-sectional analysis	Yes		N/A	N/A	N/A	Yes	
No	Yes		No		Other (specify in free text column)	Cross-sectional analysis	No		N/A	N/A	N/A	Yes	
No	Yes		No		Other (specify in free text column)	Cross-sectional analysis	No		N/A	N/A	B.117	Yes	
No	Yes		No		Other (specify in free text column)	Cross-sectional analysis	No		Not reported	Yes	Not reported	Yes	
No	No	The neutralization assay methodology unclear (could be either S fuse assay or foci reduction neutralization test (FRNT)	No		Other (specify in free text column)	Cross-sectional analysis	Other (specify in free text column)	Statistical methods used in the paper were not clearly explained (e.g. "percent increase" was used in the results but there was no explanation as to what this meant)	N/A	N/A	N/A	No	Unclear lab methods and statistical approaches
No	No	Sample size of 3 breakthrough infections, unclear whether these individuals are also included in other data	No		Other (specify in free text column)	not applicable	Other (specify in free text column)	Unclear who the 3 breakthrough infections are beyond HCWs	No	No	B.1.617.2	No	Unclear sampling and laboratory methods
No	Yes		No		Other (specify in free text column)	not reported	No	Heterogeneous age distribution, no reporting of comorbidities etc	Yes				
No	Yes		No		Other (specify in free text column)	not applicable	No		No	Unclear whether titres were taken before or after infection	Yes	No	Titres were taken "at the beginning of the outbreak", and therefore may represent titres induced by early infection in vaccinated individuals

Quality Assessment- Cohort Studies (Page 3 right)								
Yes	Yes	No	Yes	Yes	N/A	N/A	N/A	Yes
