# Supplementary material for: Does a humoral correlate of protection exist for SARS-CoV-2? A systematic review
Source: PLoS One. 2022 Apr 8;17(4):e0266852. doi: 10.1371/journal.pone.0266852 (PMC8993021; doi:10.1371/journal.pone.0266852)
Supplement: Attachment — Submitted filename: Response to reviewers 2022_03_24.docx [file pone.0266852.s004.docx]

| **Reviewer/editorial comment** | **Response** |
| --- | --- |
| 1. Please ensure that your manuscript meets PLOS ONE's style requirements, including those for file naming. | The manuscript has been reformatted according to PLOS style requirements. |
| 2. Thank you for stating the following financial disclosure:  “This work was supported by Public Health Ontario and funding from the Public Health Agency of Canada to SB.  NO”  Please state what role the funders took in the study.  If the funders had no role, please state: ""The funders had no role in study design, data collection and analysis, decision to publish, or preparation of the manuscript.""  If this statement is not correct you must amend it as needed.  Please include this amended Role of Funder statement in your cover letter; we will change the online submission form on your behalf. | The financial disclosure statement should read:  This work was supported by Public Health Ontario and funding from the Public Health Agency of Canada  A Role of Funder statement is now included in our cover letter |
| 3. Thank you for stating the following in the Acknowledgments Section of your manuscript:  “This work was supported by Public Health Ontario and funding from the Public Health Agency of Canada.”  We note that you have provided funding information that is not currently declared in your Funding Statement. However, funding information should not appear in the Acknowledgments section or other areas of your manuscript. We will only publish funding information present in the Funding Statement section of the online submission form.  Please remove any funding-related text from the manuscript and let us know how you would like to update your Funding Statement. Currently, your Funding Statement reads as follows:  “This work was supported by Public Health Ontario and funding from the Public Health Agency of Canada to SB.  NO”  Please include your amended statements within your cover letter; we will change the online submission form on your behalf. | All funding-related statements have been removed from the text and included in the Cover letter and Funding statement.  Please use the Funding Statement “This work was supported by Public Health Ontario and funding from the Public Health Agency of Canada” |
| 4. Thank you for stating the following in your Competing Interests section:  “NO”  Please complete your Competing Interests on the online submission form to state any Competing Interests. If you have no competing interests, please state ""The authors have declared that no competing interests exist."", as detailed online in our guide for authors at http://journals.plos.org/plosone/s/submit-now  This information should be included in your cover letter; we will change the online submission form on your behalf. | Cover letter now includes conflict of interest disclosure, which has been removed from the text. |
| **Reviewer #1:** The authors performed a literature review trying to determine the CoP for SARS-CoV-2 by compiling serological data on reinfections or vaccine breakthrough infections previously reported in publications or preprints. They found that while no absolute threshold for CoP can be determined up to this point, the correlate appears to be relative in a way that higher serological antibody levels in response to vaccination or infection yield greater protection against SARS-CoV-2 infection. The manuscript is very well written, but its greatest strength lies not in its findings or conclusions which are fairly generic and previously reported on, but in the vast compendium of publications they found and assessed that have attempted correlating protection against SARS-CoV-2 with serological measures. The authors are well aware of the limitations, most of which stem from the fact that there is simply too much variables in the studies – the assays and units used, the types of vaccines, and the variants causing the re-infections or breakthrough infections. The authors allude to the fact that the CoP may be different between vaccine manufacturers and variants and no single answer can be found – that is certainly the case. The authors also reported contradictory patterns from individual case studies regarding patterns between reinfection/breakthrough rates and prior antibody levels; this is not surprising as most case studies are different from the average (why they were deemed interesting as stand-alone cases) and should at most be weighted the same as a participant in a cohort study. Overall, the publication has merit as the tables serve as a useful reference guide for SARS-CoV-2 researchers and the authors are well aware of the limitations of the study. I recommend the review for publication. | We thank the reviewer for their time and their expert appraisal of our manuscript. |
| **Reviewer #2:** This paper is a review of serological correlates of protection for COVID-19 vaccines. The review is competent and complete. It reaches the conclusion that nearly everybody else has: that antibodies, particularly neutralizing, correlate with efficacy. It emphasizes that the correlate is relative, that is there is no level that corresponds to 100% efficacy. That conclusion is expected in view of published data and the fact that SARS-2 infection is primarily mucosal, similar to influenza, for which as the authors themselves say antibody is the correlate of protection although the protective level of antibody increases with titer but no absolute level is reached.. So the last line of the discussion “we do not have the tools to interpret serology with regards to protection” is silly, inasmuch as the authors describe how higher levels correlate with protection. Of course, cellular and Fc Effector immunity undoubtedly also have a role, but that does not gainsay the major role of antibody. It is also worth emphasizing that homologous levels have to be determined for each SARS-2 variant. On a trivial note, in line 162 “sera” is a plural noun and therefore “are”. | We thank the reviewer for their time and their expert appraisal of our manuscript. We have removed the last line of the discussion, and also changed line 162 (now line 163) to reflect “sera” as plural. We have also added further emphasis to the variant-specific nature of a CoP in lines 281-283. |
